# Genome-wide analysis of Ollier disease: *Is it all in the genes?*

**DOI:** 10.1186/1750-1172-6-2

**Published:** 2011-01-14

**Authors:** Twinkal C Pansuriya, Jan Oosting, Tibor Krenács, Antonie HM Taminiau, Suzan HM Verdegaal, Luca Sangiorgi, Raf Sciot, Pancras CW Hogendoorn, Karoly Szuhai, Judith VMG Bovée

**Affiliations:** 1 Department of Pathology, Leiden University Medical Center, Leiden, The Netherlands; 2 Department of Pathology and Experimental Cancer Research, Semmelweis University, Budapest, Hungary; 3 Department of Orthopaedic Surgery, Leiden University Medical Center, Leiden, The Netherlands; 4 Department of Medical Genetics, Rizzoli Orthopedic Institute, Bologna, Italy; 5 Department of Pathology, University of Leuven, Leuven, Belgium; 6 Department of Molecular Cell Biology, Leiden University Medical Center, Leiden, The Netherlands

## Abstract

**Background:**

Ollier disease is a rare, non-hereditary disorder which is characterized by the presence of multiple enchondromas (ECs), benign cartilaginous neoplasms arising within the medulla of the bone, with an asymmetric distribution. The risk of malignant transformation towards central chondrosarcoma (CS) is increased up to 35%. The aetiology of Ollier disease is unknown.

**Methods:**

We undertook genome-wide copy number and loss of heterozygosity (LOH) analysis using Affymetrix SNP 6.0 array on 37 tumours of 28 Ollier patients in combination with expression array using Illumina BeadArray v3.0 for 7 ECs of 6 patients.

**Results:**

Non-recurrent EC specific copy number alterations were found at *FAM86D, PRKG1 *and *ANKS1B. *LOH with copy number loss of chromosome 6 was found in two ECs from two unrelated Ollier patients. One of these patients also had LOH at chromosome 3. However, no common genomic alterations were found for all ECs. Using an integration approach of SNP and expression array we identified loss as well as down regulation of *POU5F1 *and gain as well as up regulation of *NIPBL*. None of these candidate regions were affected in more than two Ollier patients suggesting these changes to be random secondary events in EC development. An increased number of genetic alterations and LOH were found in Ollier CS which mainly involves chromosomes 9p, 6q, 5q and 3p.

**Conclusions:**

We present the first genome-wide analysis of the largest international series of Ollier ECs and CS reported so far and demonstrate that copy number alterations and LOH are rare and non-recurrent in Ollier ECs while secondary CS are genetically unstable. One could predict that instead small deletions, point mutations or epigenetic mechanisms play a role in the origin of ECs of Ollier disease.

## Background

Enchondroma (EC), a benign cartilage forming tumor in the medulla of the bone, is thought to be a precursor of secondary central chondrosarcoma (CS). EC develops either as a single, solitary lesion or as multiple lesions in the context of Ollier disease [1]. Ollier disease is the most common subtype of enchondromatosis and shows multiple ECs with marked unilateral predominance [1,2]. The risk of malignant transformation towards central CS is up to 35% in Ollier disease [1,3]. There is no marker that would indicate progression towards malignancy, thus there is a vital need to understand the genetics of these tumors which may help to develop markers for early diagnosis. A comprehensive understanding of the molecular events in ECs and central CS also enables the identification of possible targets for treatment [4].

While the genetics of enchondroma is poorly understood, the involvement of the EXT genes is well established in the development of solitary as well as hereditary multiple osteochondromas (MO) (OMIM 133700), benign cartilage tumors at the surface of bone [5]. The lack of EXT function seems to disturb hedgehog signalling in MO, while activated hedgehog signalling in mice seems to underlie the development of the Ollier related phenotype [4]. Heterozygous mutations in *PTH1R *are found in a small subset of Ollier patients [6-8]. It is however unclear whether these mutations in *PTH1R *are causing or modifying the disease [7], and since ~90% of Ollier patients lack *PTH1R *mutations, we aimed to study Ollier related ECs by mapping genetic changes using genomic arrays.

We hypothesized Ollier disease to be a germ-line mosaic condition due to the fact that it is non-hereditary and because of its unilateral predominance feature [3,9]. An early postzygotic mutation resulting in asymmetric involvement of skeletal structures can be expected, as was also shown for polyostotic fibrous dysplasia [10]. One could speculate that an inactivating mutation in a tumor suppressor gene, similar to EXTs in osteochondroma, would have occurred in the developing mesoderm early after gastrulation. In case of a tumor suppressor gene, later on, an additional hit may be required for the formation of ECs with subsequent genetic changes causing progression towards central CS. We tested this hypothesis using high-resolution SNP array combined with expression array on DNA derived from tumor tissue and, whenever available, normal DNA from Ollier patients.

SNP arrays provide an excellent possibility for large-scale, genome-wide, high-resolution analysis of both DNA copy number alterations (CNA) and loss of heterozygosity (LOH) in cancer cells. It also provides a feasible means of detecting genotyping alterations in the tumors of individual patients and, in principle enables the identification of new areas with common allelic imbalance that could harbor potential tumor suppressor genes which helps in the identification of novel candidate genes affected by genomic abnormalities [11,12]. In the present study, we used Affymetrix Genome-Wide Human SNP Array 6.0 to obtain a comprehensive registry of genetic aberrations in 37 tumors of 28 patients with Ollier disease and correlate it with gene expression using Illumina Human-6 v3 Expression Array and qRT-PCR and protein expression using tissue microarray (TMA). Based on the obtained genomic profiles with limited and non-recurrent genetic changes in Ollier ECs, we conclude that they are genetically heterogeneous and that the reported CNA in this study are likely to be secondary random events in ECs.

## Materials and methods

### Patient materials and reference samples

Fresh frozen tissues from 37 tumors from 28 patients diagnosed with Ollier disease were collected from the EuroBoNet consortium (http://www.eurobonet.eu) (Table 1): Leiden University Medical Center, The Netherlands (12 tumors), Leuven University, Belgium (5 tumors), Rizzoli Institute, Italy (6 tumors), Royal Orthopaedic Hospital, United Kingdom (7 tumors), Lund University, Sweden (2 tumors), Netherlands Committee on Bone Tumors, The Netherlands (2 tumors), Heidelberg University, Germany (1 tumor), University of Ghent, Belgium (1 tumor) and Groningen University Medical Center, The Netherlands (1 tumor). All samples were derived from primary tumors, not from recurrent tumors, and all were graded according to Evans et al [13]. Diagnoses were originally made in the multidisciplinary teams of the centers of origin. The histology was revised and representativity was assessed on the available paraffin or frozen tissue by one experienced bone tumor pathologist. For SNP array analysis, 14 Ollier ECs, and 23 Ollier CS (13 grade I, 8 grade II, 2 grade III) from 28 Ollier patients were used. As controls, normal DNA derived from fresh frozen muscle tissue (n = 3), peripheral blood lymphocytes (n = 4) or saliva (n = 4) was available for 11 Ollier patients and 3 patients with unrelated bone diseases. We used blood lymphocyte DNA from 12 healthy controls and 1 HapMap sample. We also isolated DNA from saliva for 3 of these controls to validate the use of saliva DNA in this study. Twenty eight of these thirty controls and DNA from 10 additional HapMap samples were used in MLPA. RNA from 4 articular cartilage, 2 growth plates and 7 ECs was used for expression array and 3 articular cartilage, 2 growth plates and 8 ECs were used in qRT-PCR.

**Table 1:** Clinicopathological data of the Ollier patients

*Patient ID*	*Sample*	*Tumor Grade*	*Tumor location*	*Gender*	*Age*	*Application*
17	L1083	CS I	metacarpal	M	48	1,3
17	L2218	CS I	phalanx	M	49	1,3
20	L286*	CS II	femur	F	23	1,3
21	L204*	CS I	femur	M	26	1,3
21	L253*	CS I	tibia	M	26	1,3
22	L206	EC	phalanx	F	26	1,2,3,4
22	L910	EC	phalanx	F	16	1,2,3,4
23	L813	CS II	humerus	M	68	1,3
25	L1251*	EC	phalanx	M	15	1,2,3,4
25	L2220*	EC	metacarpal	M	14	1,3,4
26	L1974	CS II	scapula	M	48	1,3
27	L1975*	CS II	femur	M	31	1,3
28	L1976*	CS II	tibia	M	41	1,3
29	L1977*	CS I	tibia	M	41	1,3
29	L1978*	EC	foot	M	38	1,3
30	L1980	CS II	knee	F	63	1,3
31	L810	CS III	unknown	M	-	1,3
33	L1685	CS I	pubic bone	F	23	1,3
34	L1687	CS I	phalanx	M	18	1,3
34	L1686	EC	phalanx	M	18	1,2,3,4
35	L2386	CS I	phalanx	F	13	1,3
36	L2463*	EC	tibia	F	12	1,3
38	L1629	EC	unknown	M	36	1,3
38	L1630	EC	Iliac bone	M	36	1,2,3,4
42	L2098	CS II	humerus	F	15	1,3
43	L2099	CS II	humerus	F	49	1,3
47	L2103a	EC	phalanx	M	39	1,2,3,4
47	L2103b	CS I	phalanx	M	39	1,3
48	L2104a	CS III	tibia	M	35	1,3
48	L2104b	EC	femur	M	35	1,3
50	L2221*	CS I	femur	F	42	1,3
52	L1513*	CS I	femur	F	23	1,3
54	L1490*	EC	phalanx	F	12	1,3
61	L2205	EC	ilium	M	6	1,3
64	L1683	EC	metacarpal	F	29	1,2,3,4
68	L2280*	CS I	acromion	F	24	1,3
69	L2513	CS I	pelvis	M	33	1,3

* Normal DNA available enabling paired analysis. Application - 1: sample used for SNP array, 2: expression array, 3: MLPA and 4: qRT-PCR.

Five tissue micro array (TMA) blocks containing 86 tumors were constructed, of which 65 were Ollier related and 21 were solitary central tumors (ECs and CS) from both the EuroBoNet and the European Musculoskeletal Oncology Society (EMSOS) networks (Table S1, Additional file 1): Leiden University Medical Center, The Netherlands (27 tumors), Rizzoli Institute, Italy (12 tumors), Copenhagen University, Denmark (9 tumors), University clinic of Orthopaedic Surgery and Medical university of Graz University, Austria (6 tumors), Bern University, Switzerland (5 tumors), University of Navarra, Spain (4 tumors), Netherlands Committee on Bone Tumors (21 tumors), Istanbul University, Turkey (2 tumors). All the samples were obtained according to the ethical guidelines of the host institution. Samples were coded and all procedures were performed according to the ethical guidelines "Code for Proper Secondary Use of Human Tissue in The Netherlands" (Dutch Federation of Medical Scientific Societies).

### DNA and RNA isolation

Tumor samples were selected that contained more than 80% of tumor cells, as estimated on haematoxylin and eosin-stained frozen sections. Most of the samples were macro dissected and L2099 sample was micro dissected to enrich the tumor percentage [14]. DNA from fresh frozen tissue was isolated using the wizard genomic DNA purification kit (Promega, Madison, WI), according to the manufacturer instructions. Blood DNA was isolated as described by Miller et al [15]. Saliva DNA was isolated using the Oragene DNA kit (DNA Genotek Inc Ontario, Canada) according to the protocol provided by the supplier and DNA was precipitated using sodium acetate precipitation. DNA concentration was quantified spectrophotometrically using Nanodrop ND-1000 (Thermo Fisher Scientific, Waltham, MA, USA) and the fragment sizes were determined on 1% agarose gel.

RNA isolation from the fresh frozen tissue was performed as described previously [16]. RNA concentration was measured spectrophotometrically and the fragment sizes were determined by RNA 6000 Nano LabChip kit using Agilent 2100 Bioanalyzer (Santa Clara, CA, USA). DNA and RNA from all the samples were good enough to continue with the experiment.

### SNP Array and Data Analysis

We used the Affymetrix Genome-Wide Human SNP Array 6.0. Genomic DNA preparation, labeling and hybridization were performed according to Affymetrix's recommended protocols (Affymetrix, Santa Clara, CA, USA). Then, arrays were scanned with GeneChip™ GSC3000 7G Whole-Genome Association System (Affymetrix). Overall hybridization quality was estimated by the genotype call rate using the Birdseed genotype calling algorithm in Genotyping Console (version 3.0.2, Affymetrix). Average call rate was 97.83%. To analyze the data we used statistical language R version 2.8 and Nexus software version 4.1 (BioDiscovery, CA, USA). We did not use HapMap samples as baseline in this study to avoid the bias for the experiment performance at different labs, batch effect and hybridization quality. The analysis was performed on a subset (30 controls, 14 ECs and 23 CS of Ollier patients, Table 1) of a larger experiment of 92 samples including samples unrelated to Ollier disease to achieve a larger set of common controls. Than we performed copy number analysis using 92 samples as a baseline in R software and only 29 control samples of high quality as a baseline in Nexus software. Results that we obtained using different softwares and different baselines were comparable. We used CEL files in R software. For the genomic analysis using R, we did genotyping using the CRLMM algorithm in the Oligo package [17], copy number analysis using the aroma affymetrix package [18], and we constructed LAIR plots to visualize regions of LOH and allelic imbalance [19]. Chromosome-X was not analyzed to avoid gender-related issues [20]. In Nexus, we performed copy number analysis using CNCHP log-ratio files generated by genotyping console using 29 controls as a baseline. Hidden Markov model (HMM) based SNP-FASST segmentation was used to identify aberrant genomic regions. Here we considered at least 5 probes for each segment. The data discussed in this publication have been deposited in NCBI's Gene Expression Omnibus (GEO) database (http://www.ncbi.nlm.nih.gov/geo/ accession number GSE22965).

### Multiplex Ligation-Dependent Probe Amplification (MLPA)

MLPA was used to confirm copy number gains and losses found within the selected candidate genes by SNP array. MLPA probes were designed using NCBI Build 36.1. We used two probes for *TCRA *and one probe for *POU5F1, ANKS1B, FAM86D *and *PRKG1*. Probe sequences can be obtained upon request. MLPA was performed as described previously [21]. Sample series of SNP array (Table 1) and in addition to that DNA from ten HapMap samples was used. Data analysis was done using SoftGenetics GeneMarker version 1.70. We set 1.2 and 0.8 as a threshold to detect the gains and losses respectively.

### Expression Array and Data Analysis

In total, we analyzed 7 ECs while 2 growth plate and 4 articular cartilage samples served as a control (Table 1). Expression array was performed using Illumina Human-6 v3.0 Expression BeadChips (Illumina Inc., San Diego, CA). For Illumina BeadArray assay, cRNA was synthesized with an Illumina RNA Amplification Kit (Ambion, Austin, TX, USA), purified, labeled and hybridized as per the manufacturer's instructions. Then, arrays were scanned with Illumina BeadArray reader (500GX, Illumina) and scanned images were imported in BeadStudio software version 3.1.3.0 (Illumina). For the expression array, unprocessed data were collected from BeadStudio and processed using VST [22] and quantile normalization with the R bead array package [23]. The processed data was analyzed using Linear Models for Microarray Data (LIMMA) analysis which uses moderated t-test to detect differentially expressed genes between two groups by taking into account natural variance within these groups and correcting for multiple testing using false discovery rate [24].

### Real-time quantitative Reverse Transcriptase PCR (qRT-PCR)

qRT-PCR was used to confirm the results obtained by the integration of SNP and expression array. Here we have used 5 controls (2 growth plates and 3 articular cartilage) and 8 ECs (Table 1). RNA used for expression array was also used for the qRT-PCR. cDNA was synthesized using 1 μg of total RNA with AMV reverse transcriptase (Roche Applied Science, Almere, The Netherlands) in combination with oligo-dT and random hexamer priming and qPCR was performed as described elsewhere [25]. Primer details can be provided upon request. Expression of the genes of interest (*FAM86D, POU5F1 *and *ANKS1B*) was normalized by geometric averaging of multiple internal control genes using the geNorm program [26]. Out of four normalization genes the best three were selected within this program: *GPR108*, *SRPR *and *TBP*. Relative quantification was performed using standard curves, followed by adjustment with the normalization factor calculated by geNorm program [25]. The average relative expression of gene of interest in ECs was compared to controls. To see the EC specific gene expression changes, relative expression of ECs with change in copy number was compared with ECs without change in copy number.

### Tissue Microarray (TMA)

Five TMA blocks were constructed using TMA master (Zeiss, 3D Histech, Hungary) and each block contain maximum 70 cores including seven control tissues (growth plate, articular cartilage, breast carcinoma, prostate, colon, skin and tonsil) for orientation purpose. In total 5 TMA blocks contain 86 tumors, of which 65 Ollier related and 21 solitary ECs and CS (Table S1, Additional file 1). The TMA blocks contain 2-mm cores of each tumor in triplicate.

### Immunohistochemistry

Tissue sections were cut from TMA blocks and dried overnight at 60˚C. Slides were kept in Xylol for 20 minutes. Immunohistochemistry was performed as described earlier [27]. Detailed information on the antibodies used to check protein expression of POU5F1 and NIPBL are given in table S2, additional file 1. We used power vision (poly-HRP-GAM/R/R, Immunologic) as a secondary antibody. Visualization was carried out with liquid Dab^+ ^substrate chromogen system (DAKO, Glostrup, Denmark). As a negative control primary antibody was omitted. Immunostained TMA slides were scanned using a high resolution Mirax Desk instrument (Zeiss, Mirax 3D Histech, Hungary) and scored independently by two observers (JVMGB and TCP) with the Mirax viewer TMA module software version 1.1.12 (Zeiss) and discrepancies were discussed. In brief, the intensity (0 = no staining, 1 = weak, 2 = moderate, 3 = strong) and percentage of positive tumors cells (0 = 0%, 1 = 1-24%, 2 = 25-49%, 3 = 50-74%, 4 = 75-100%) were assessed. Cores with a negative internal control and loss of tissue were excluded from the analysis. A sum score ≥ 4 was considered positive. Statistical analysis was done using Pearson Chi-Square test in SPSS (version 16.0, Chicago, Illinois, USA).

## Results

### Genome-wide detection of copy number alterations and loss of heterozygosity using SNP array

**Figure 1:**
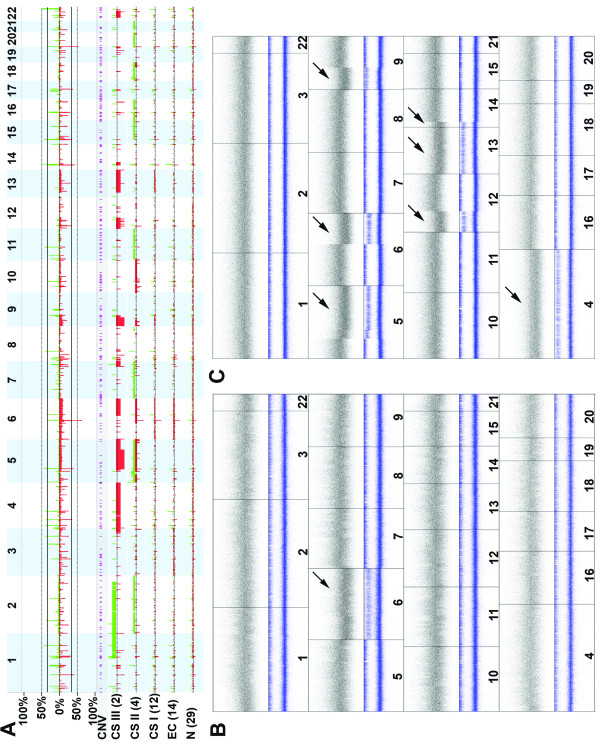
**Genome-wide copy number alterations in all 22 chromosomes**. A) Copy number alterations in controls, Ollier enchondromas (ECs) and chondrosarcoma (CS) grade I, II, III. The upper panel shows genome-wide frequency plots of gains and losses in 29 controls and 32 Ollier tumors. Gains are plotted in green above 0% baseline and losses are plotted in red below 0% baseline. The X-axis corresponds to the genomic region from chromosomes 1 to 22 and the Y-axis represents the percentage of gains and losses of all selected samples at the specific location in genome. The lower panel shows frequency plot of 29 controls, 14 ECs, 12 CS I, 4 CS II and 2 CS III. The number and size of genomic alterations increases with increasing tumor grade. Enchondromas and control samples show a comparable number and size of genomic alterations, which can be attributed to common copy number variation. B) An example of copy number alterations in Ollier enchondroma (L206). This figure shows copy number alterations in all 22 chromosomes of enchondroma (L206). The black band indicates the number of copies of the chromosomes. The blue bands show the unpaired LAIR value. The lower band of this contains the originally uninformative homozygous SNPs. The top band indicates heterozygous informative SNPs. With LOH or imbalances between the alleles, the position of this band will decrease. As a loss of chromosome 6 these heterozygous SNPs are becoming homozygous showing the LOH. C) An example of copy number alterations in Ollier chondrosarcoma grade III (L810). Copy number loss with LOH is present at chromosome 4, 5q, 6q, 9p, 12p, 13 and 14q.

Samples L1975, L1974, L1980, L813, L1083 and L286N were excluded from further analysis because of quality issues and low call rates. All samples including 29 controls and 32 tumors (14 ECs, 18CS) showed DNA copy number aberrations mainly restricted to known variable regions (Figure 1)[28,29]. CNA were more frequently found in CS II and CS III as compared to ECs and CS I (Figure 1). The number of copy number changes in saliva and blood DNA were comparable.

#### Genetic alterations in Ollier Enchondromas

We used paired analysis which is based on the comparison of tumor and corresponding normal DNA (available for 5 ECs from 4 patients) to study LOH and CNA. Although sequences of homozygous SNPs were identified that could indicate LOH, these same sequences were also observed in the corresponding normal sample. We could not find any LOH in these 5 ECs using both R and Nexus softwares. We have identified 7 EC specific CNA by paired approach (Table 2). Selection of candidate genes *TCRA*, *ANKS1B *and *PRKG1 *for further validation is based on copy number change in minimum 10 probes within the gene.

**Table 2:** Paired copy number alterations in Ollier enchondromas

*Patient ID*	*Sample*	*Cytoband*	*Copy number event in EC*	*Region*
29	L1978	14q11.2*	gain	TCRA
25	L2220	12q23.1*	loss	ANKS1B
25, 54	L2220, L1490	10q11.22	loss	intergenic region
54	L1490	1p31.3	loss	intergenic region
54	L1490	2q11.2	gain	intron of VWA3B
54	L1490	5q13.2	loss	intergenic region
54	L1490	10q11.23*	gain	intron of PRKG1

* Candidate regions selected for further validation based on minimum 10 affected probes within the gene.

Unpaired analysis revealed absence of LOH in the majority of ECs. We confirmed the loss of chromosome 6 with LOH in L206 (Figure 1B) which was in agreement with the results published previously by our group using array CGH [30] proving validity of the assay. Also loss of one copy of chromosome 3 and 6 with LOH was found in L1683. These results were confirmed using R and Nexus software. Furthermore, an unpaired approach (29 controls as a baseline) was used to find most common copy number gains and losses in at least 5 out of 14 ECs (Table S3, Additional file 1) using Nexus. None of these were confirmed in paired comparison which suggests that they are not tumor specific changes. When ECs are located in the phalangeal bones, cellularity is increased [1]; we could not find differences between ECs or CSI of the hand versus those of long bones at the genomic level although the sample sizes are small.

**Figure 2:**
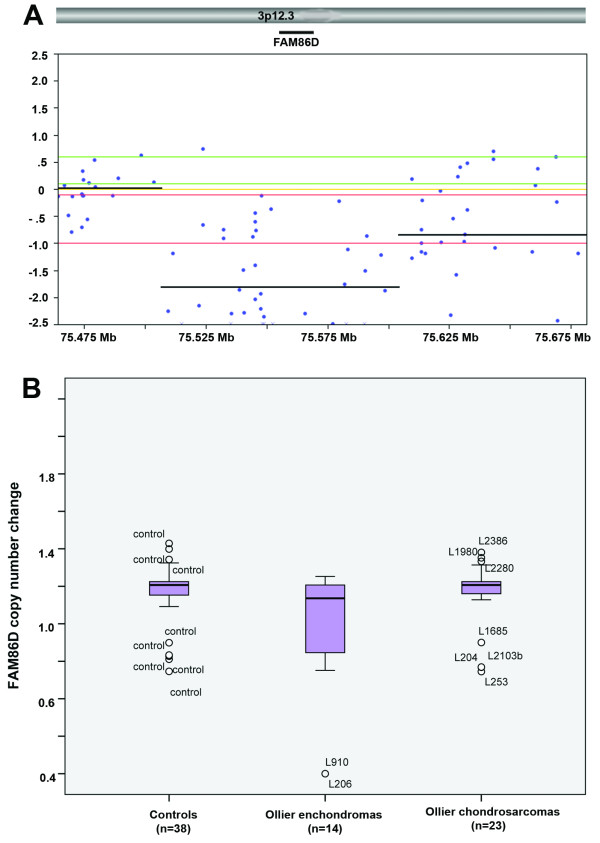
***FAM86D***. A) Homozygous loss of *FAM86D *in L206. A 200 Mb region containing the *FAM86D *gene at 3p12.3 is shown (X-axis). The gene lies in a ~100 Mb homozygous deleted region, within a larger area of hemizygous deletion. The individual copy number probes are shown as the log ratio of the intensity and zero is two copies. The horizontal lines are segments with identical copy number as identified by the HMM SNP-FASST algorithm. B) MLPA for 38 controls, 14 Ollier enchondromas (ECs) and 23 Ollier chondrosarcomas (CS). The Y-axis shows ratio profile and 1.0 indicates two copies of a given chromosomal locus. Homozygous copy loss of the *FAM86D *as shown by SNP array was confirmed in L206 and L910. Most of the controls and tumors show either two copies or hemizygous loss of this gene. Note that only outliers are displayed with ID numbers.

Homozygous deletion of *FAM86D *at chromosome 3p12.3 was found in two ECs in one patient (L206 and L910) and selected for further validation (Figure 2). Interestingly both ECs were located in different digits of the hand. It was not possible to get normal DNA from the same patient to investigate tumor specific loss of *FAM86D*.

#### Genetic alterations in Ollier chondrosarcomas

Ollier CS showed large genomic alterations and LOH at various locations in the genome (Table 3, Figure 1C). Frequent recurrent CNA involves chromosomes 3p, 5q, 6q and 9p.

**Table 3:** Genetic alterations in Ollier chondrosarcomas

*Sample*	*Tumor Grade*	*Gain*	*Loss*	*LOH*	*Copy neutral LOH*
L2218	CS I	-	-	-	-
L204	CS I	1q	3p,4q	3p,4q	-
L253	CS I	1q	6p,6q,9p,12q,13	6p,6q,9p,12q,13	-
L1977	CS I	-	-	-	-
L1685	CS I	-	2q	2q	-
L1687	CS I	3p,7q,8q	-	-	-
L2386	CS I	-	6p,11q	-	-
L2103b	CS I	-	-	-	-
L2221	CS I	14q,17q	-	-	-
L1513	CS I	-	-	-	-
L2280	CS I	-	-	-	-
L2513	CS I	-	-	-	-
L286	CS II	5p,11p,11q,18p	3p,5p,5q,6q,9p,11p,18p,18q	3p,5p,6q,9p,11p,18p,18q	-
L1976	CS II	8q	-	8q	-
L2098	CS II	2,5,7,15,16,17q,18,20,21	10,17q	10,17q	11
L2099	CS II	-	-	-	-
L810	CS III	-	4,5q,6q,9p,12p,13,14q	4,5q,6q,9p,12p,13,14q	-
L2104a	CS III	1q,2p,2q,12p,14q	3q,5q,7q,9p,12p, 22	3q,5q,7q,9p,12p,22	2q

Some of the regions with loss do not show loss of heterozygosity (LOH). This could be explained by the loss of alleles in an aneuploid background.

### Verification of gene copy number alterations by MLPA

To validate regions identified by SNP arrays, we performed MLPA on 37 Ollier tumors (Table 1) and 38 controls for the candidate genes *FAM86D, TCRA*, *ANKS1B *and *PRKG1 *(Table 4). In addition, a probe was designed at *POUF51 *which is at chromosome 6p21.31 in order to confirm loss of chromosome 6 in L206 and L1683. We have used 38 controls as a baseline. Results of validation experiments are summarized in Table 4. In short we confirmed three (*FAM86D*, *PRKG1 *and monosomy 6) out of five candidate regions. We confirmed the homozygous loss of *FAM86D *in two ECs of the same patient (Figure 2). Gain at *PRKG1 *in L1490 is confirmed which seems to be EC specific in this case but was not found in other ECs and CS. Loss at chromosome 6 using probe at *POU5F1 *was confirmed in L206 while loss was not confirmed in L1683 at given thresholds. *TCRA *region at chromosome 14q11.2 is known as a highly polymorphic region [31] and its frequent rearrangements are observed in blood lymphocytes [32]. We used majority of blood samples as a baseline and found copy number gain in all ECs at *TCRA *as a result of SNP array unpaired analysis. Paired analysis with SNP array showed copy number gain at *TCRA *in L1978 and was not confirmed with MLPA. Indeed this gain was due to loss in the corresponding normal DNA. Loss at *ANKS1B *in L2220 was not confirmed by MLPA at given thresholds however the peak was lower in L2220 compared to corresponding normal.

**Table 4:** Summary of validation experiments for the candidate genes

*Candidate genes*	*Technique used*	*Summary of validation*
*PRKG1*	MLPA	No copy number change in controls. Gain in tumor (1/37)
*FAM86D*	MLPA, qRT-PCR	Loss in controls (5/38), gain in controls (1/38), HMloss in tumors (2/37) loss in tumours (6/37). Lower m-RNA expression in ECs
*POU5F1*	MLPA, qRT-PCR, IHC	Gain in controls (3/38), loss in tumors (4/37), gain in tumors (2/37). Its mRNA and protein expression was absent in tumors
*ANKS1B*	MLPA, qRT-PCR	No copy number changes in controls and tumors. Lower m-RNA expression in L2220 compared with other 7ECs
*TCRA *_probe1	MLPA	Loss in controls (4/38), gain in tumors (2/37)
*TCRA *_probe2	MLPA	Loss in controls (4/38), gain in tumors (2/37)
*NIPBL*	IHC	30% Ollier tumors showed protein expression

### Expression array and its integration with copy number alterations

Expression array was performed using 7 ECs and 6 controls with Illumina Human-6 v3.0 (Table 1). We performed function based analysis by integrating the gene expression results with SNP array results. In total 1044 genes were differentially expressed in ECs compared to controls (adj. p-value < 0.01, Table S4, Additional file 1). We considered all up regulated genes (881 genes, adj. p-value < 0.01) with presence of gain in at least one EC and all down regulated genes (163 genes, adj. p-value < 0.01) with presence of loss in at least two ECs. We found *NIPBL *which was gained as well as up regulated while *POU5F1 *that was lost as well as down regulated in ECs compared to controls. The same pattern was found in CS.

### Validation of integration approach of SNP and expression array using qRT-PCR

Using qRT-PCR, we confirmed lower expression of *ANKS1B*, *FAM86D *and *POU5F1 *(Table 4). L2220 showed one copy loss of *ANKS1B *and its expression was decreased in this EC as compared to the average of relative expression of other 7 ECs however there is no difference in the expression comparing all ECs versus controls. SNP array results revealed homozygous loss in L206, L910 and one copy loss in L2103 for *FAM86D*. *FAM86D *expression was decreased in ECs compared to controls. There was no expression of *POU5F1.*

### Protein expression

**Figure 3:**
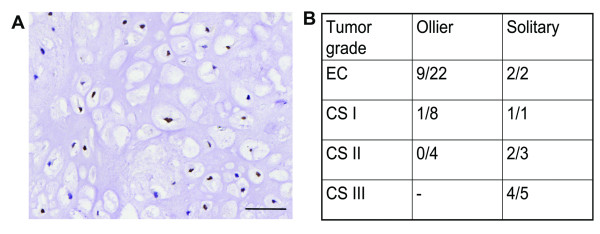
**NIPBL protein expression**. A) Example of nuclear expression of *NIPBL *in Ollier enchondroma (400 times magnification). B) Number of Ollier and solitary tumors with nuclear NIPBL expression.

Using a TMA we demonstrated that POU5F1 protein expression was absent in all Ollier tumors which is in line with the expression array and qRT-PCR results. For NIPBL, 30% of Ollier tumors were positive (Figure 3, Table 4). Only 34/65 Ollier and 11/21 solitary tumors could be analyzed since cores from the rest of the tumors were lost during the immunohistochemistry. There was no significant difference in the expression of NIPBL within the tumors of different grades in Ollier disease (Pearson Chi-Square, p-value = 0.1).

## Discussion

The origin of both solitary and Ollier related ECs is largely unknown. To address this, we performed genome-wide analysis of ECs occurring in non-hereditary Ollier disease. Since these tumors are polyostotic, with a unilateral predominance, manifesting early in life, we postulated that there may be a germ-line mosaicism. We attempted to find causative genes for ECs of the Ollier disease using a high-resolution SNP array containing 1.8 million markers combined with expression array and obtained comprehensive genetic profiles of 37 Ollier disease related tumors. This is the first and largest genome-wide molecular study on Ollier disease reported so far, which was possible through the collaboration of many different institutes within the EuroBoNeT Network and the European Musculo-Skeletal Oncology Society (EMSOS).

In general, the obtained genomic profiles showed absence of large genetic aberrations in Ollier ECs except loss of chromosome 6 in two ECs from two unrelated Ollier patients. Small non-recurrent genetic changes combining the SNP and expression array at *FAM86D*, *PRKG1*, *ANKS1B, NIPBL *and *POUF51 *were found in ECs. Most of these genes are not known to play an important role in cartilage formation. We found homozygous loss of *FAM86D *in two ECs of the same patient. Function of *FAM86D *is still unknown. We confirmed intronic gain at *PRKG1 *in one EC, which is involved in fatty acid metabolism [33]. We found loss at *ANKS1B*, while overexpression of *ANKS1B *is reported in pre-B cell acute lymphocytic leukaemia [34]. Gain at *NIPBL *was found in L2205 while at the protein level only 30% of Ollier ECs and CS expressed NIPBL. Inactivating mutations in *NIPBL *are associated with Cornelia de Lange syndrome and one of the characteristic features of this syndrome is reduction in limb growth (OMIM 122470). Loss of *POU5F1 *was found in two ECs with monosomy at chromosome 6 and its mRNA and protein expression was absent in all Ollier and solitary ECs and CS. The transcription factor *POU5F1 *(OCT3/4) is involved in regulating pluripotency and is normally expressed during early embryogenesis in embryonic stem and germ cells [35].

Here we studied extensively candidate genes obtained from paired analysis of enchondromas. Normal DNA was available from 4 Ollier patients enabling paired analysis. Despite the low number of paired samples our data suggest that no common CNA are associated with EC development. Extending the analysis with the unpaired samples we could not see any common CNA in all ECs. All aberrations we obtained in at least 5 out of 14 ECs are reported as common copy number variants in DGV database (http://projects.tcag.ca/variation/). Loss of chromosome 6 was the only recurrent change in ECs of two unrelated Ollier patients. Therefore, relatively small numbers of copy number alterations that we found per ECs are more likely to be secondary random genetic changes. Although SNP array technology is a powerful analysis tool, it can not detect balanced chromosomal translocations, inversions and whole-genome ploidy changes. However, balanced translocations and inversions have not been reported for the Ollier tumors in the literature so far [2].

Previously, *PTH1R *was reported to be the gene causing Ollier disease [6]. However, in subsequent studies it was shown that *PTH1R *is only mutated in a sub group of patients (~10%) decreasing receptor function to ~70%, suggesting that it may contribute to the disease but is probably not causative [7]. PTH1R is a key player within the IHH pathway which is crucial for endochondral ossification. The presence of known point mutations (R150C, R255H, G121E and A122T) in *PTH1R *was excluded in the present series (unpublished data). Also, we could not find a deletion or LOH at the 3p21.31 region harboring the *PTH1R *gene. Recently, inactivating mutations in *PTPN11 *are reported in another enchondromatosis subtype called metachondromatosis in which multiple ECs are combined with osteochondroma-like lesions [2,36]. In our series we could not detect any CNA or LOH at *PTPN11 *by SNP array. Also, expression of *PTPN11 *was not decreased in ECs as compared to controls in expression array.

Large gains, losses and LOH were seen more often in Ollier CS as compared to ECs, which is in line with increased genetic instability and aneuploidy seen in solitary central chondrosarcoma progression. Most common CNA involve 3p, 5q, 6q and 9p in Ollier CS. However, we could not detect recurrent CNA in all Ollier CS that could have been responsible for malignant transformation of ECs. For Ollier CS, a deletion at the short arm of chromosome 1 [37], LOH at *RB1 *at chromosome 13 and p15/p16 loci at the short arm of chromosome 9 [38] were reported in single cases each. Our results show very few copy number alterations in ECs and an increased number of variable genomic alterations in CS. This is in support of the multistep model for CS development [39].

In conclusion, we present genome-wide copy number and expression profiles of the largest international series of Ollier ECs and CS reported so far. We show absence of recurrent CNA and LOH in majority of ECs suggesting that instead point mutations or other copy number neutral structural changes (inversions, insertions, balanced translocations) or deletions below the resolution of this platform involving a single or a few exons only [40] might have an important role in EC pathogenesis. This opens the possibility to study these tumors further using a next generation sequencing approach. An increased number of genetic alterations are found in Ollier CS, supporting the multistep genetic progression model.

## Abbreviations

EC: Enchondroma; CS: Chondrosarcoma; SNP: Single nucleotide polymorphism; LOH: Loss of heterozygosity; CAN: Copy number alterations; TMA: Tissue microarray; MLPA: Multiplex Ligation Dependent Probe Amplification

## Competing interests

The authors declare that they have no competing interests.

## Authors' contributions

The study was designed by TCP, JVMGB, PCWH, JO and KS. Data analysis was done by TCP and JO. Tissue microarray was constructed by TCP and TK. RS, LS, AHMT, SHMV were responsible for acquisition of patient material and patient data. The manuscript was written and approved by all the coauthors.

## Supplementary Material

Additional file 1 **Table S1, Table S2, Table S3, Table S4**. Table S1 - Clinicopathological data of 86 tumors used in TMA - * Gender information was not available for four Ollier patients. Table S2 - Antibody information Table S3 - Unpaired copy number changes in 35% of EC (min. 5 out of 14) - All the copy number gains and losses that we found in more than four ECs are an indicative of known copy number variation in DGV database of genomic variants. Table S4 - List of up and down regulated genes in enchondromas compared to controls using expression array (adj. p-value < 0.001) Click here for file
