# Supplementary material for: Genome-wide analysis of Ollier disease: Is it all in the genes?
Source: Orphanet J Rare Dis. 2011 Jan 14;6:2. doi: 10.1186/1750-1172-6-2 (PMC3027091; doi:10.1186/1750-1172-6-2)
Supplement: Additional file 1 — Table S1, Table S2, Table S3, Table S4. Table S1 - Clinicopathological data of 86 tumors used in TMA - * Gender information was not available for four Ollier patients. Table S2 - Antibody information Table S3 - Unpaired copy number changes in 35% of EC (min. 5 out of 14) - All the copy number gains and losses that we found in more than four ECs are an indicative of known copy number variation in DGV database of genomic variants. Table S4 - List of up and down regulated genes in enchondromas compared to controls using expression array (adj. p-value < 0.001) [file 1750-1172-6-2-S1.DOC]

**Table S1: Clinicopathological data of 86 tumors used in TMA**

|  | *Ollier* | | *Solitary* |
| --- | --- | --- | --- |
| Total number of patients | | 43 | 21 |
| Total number of tumor samples | | 65 | 21 |
| EC | | 39 | 6 |
| CS I | | 17 | 4 |
| CS II | | - | 5 |
| CS III | | 9 | 6 |
| Male:Female | | 20:19* | 13:8 |
| Median age at diagnosis years (range) | | 19 (3-63) | 55 (29-84) |

* Gender information was not available for four Ollier patients.

**Table S**2: Antibody information

| *Protein* | *Antibody Identification* | *Dilution* | *Antigen retrieval* | *Positive control* | *Staining* | *Company* |
| --- | --- | --- | --- | --- | --- | --- |
| NIPBL | 250133 | 1:800 | citrate, microwave | normal lung | nuclear | Abbiotec |
| POU5F1 | ab73099 | 1:3 | citrate, microwave | testicular tumor | cytoplasm | Abcam |

**Table S3: Unpaired copy number changes in 35% of EC (min. 5 out of 14)**

| *Number of ECs* | *Region* | *Cytoband* | *Copy number event* | *Genes* | *% of CNV overlap* |
| --- | --- | --- | --- | --- | --- |
| 5 | chr1:0-218,741 | p36.33 | gain | 6 | 99.8 |
| 5 | chr1:142,693,888-143,978,071 | q21.1 | gain | 11 | 100 |
| 5 | chr1:16,758,739-16,878,452 | p36.13 | gain | 3 | 100 |
| 5 | chr1:194,993,359-195,068,330 | q31.3 | gain | 2 | 100 |
| 5 | chr1:25,468,751-25,519,541 | p36.11 | gain | 1 | 100 |
| 7 | chr4:69,056,055-69,170,240 | q13.2 | gain | 1 | 100 |
| 5 | chr7:142,155,398-142,171,867 | q34 | gain | 1 | 100 |
| 5 | chr8:12,286,694-12,287,223 | p23.1 | gain | 1 | 100 |
| 5 | chr9:44,889,904-47,006,984 | p11.2 - p11.1 | gain | 3 | 100 |
| 5 | chr9:65,336,138-70,168,361 | q12 - q13 | gain | 23 | 100 |
| 5 | chr10:46,401,243-46,481,060 | q11.22 | gain | 1 | 100 |
| 5 | chr11:18,905,781-18,918,566 | p15.1 | gain | 1 | 100 |
| 5 | chr11:5,745,410-5,759,390 | p15.4 | gain | 1 | 100 |
| 6 | chr11:55,125,250-55,213,752 | q11 | gain | 4 | 100 |
| 5 | chr14:18,072,112-19,492,811 | q11.1 - q11.2 | gain | 9 | 92.32 |
| 5 | chr15:18,671,839-20,157,067 | q11.2 | gain | 11 | 100 |
| 5 | chr16:32,859,978-33,530,318 | p11.2 | gain | 4 | 100 |
| 5 | chr16:34,320,629-34,620,525 | p11.2 - p11.1 | gain | 2 | 100 |
| 5 | chr19:47,950,927-48,049,905 | q13.31 | gain | 2 | 100 |
| 5 | chr20:1,509,277-1,530,137 | p13 | gain | 1 | 100 |
| 5 | chr21:9,985,800-10,195,652 | p11.2 - p11.1 | gain | 6 | 100 |
| 6 | chr8:7,225,806-7,830,758 | p23.1 | loss | 26 | 100 |
| 5 | chr10:27,265,953-27,269,233 | p12.1 | loss | 1 | 100 |
| 5 | chr14:18,072,112-19,495,051 | q11.1 - q11.2 | loss | 9 | 92.32 |
| 5 | chr20:1,509,277-1,543,885 | p13 | loss | 1 | 100 |

All the copy number gains and losses that we found in more than four ECs are an indicative of known copy number variation in DGV database of genomic variants.

**Table S4: List of up and down regulated genes in enchondromas compared to controls using expression array (adj. p-value<0.001)**

| *Gene* | *Probe ID* | *Log fold change* | *Adjusted p-value* | *Up or down regulated in ECs compared to controls* |
| --- | --- | --- | --- | --- |
| RPL5 | 830609 | 1.498288353 | 1.23E-05 | up |
| SEPHS1 | 5700612 | 0.882184 | 1.44E-05 | up |
| FBXL7 | 6980056 | 0.411650382 | 2.24E-05 | up |
| PENK | 6220019 | 6.282102003 | 2.87E-05 | up |
| HNRPA1L-2 | 7320424 | 1.445469169 | 2.87E-05 | up |
| KLHDC2 | 2810364 | 1.249636151 | 2.87E-05 | up |
| RPS15A | 2370193 | 1.70815712 | 3.15E-05 | up |
| PSAT1 | 4850674 | 3.132269511 | 3.27E-05 | up |
| NARS | 5220653 | 1.472789211 | 3.27E-05 | up |
| DDOST | 6450605 | 1.169205597 | 3.27E-05 | up |
| SUB1 | 1660661 | 0.812277392 | 3.81E-05 | up |
| MORF4L1 | 4900343 | 1.892734965 | 5.16E-05 | up |
| SERP1 | 1770541 | 1.250963064 | 5.85E-05 | up |
| HNRPDL | 2570358 | 0.910611746 | 5.85E-05 | up |
| RGMB | 840553 | 1.178803561 | 6.55E-05 | up |
| ARPP19 | 6620356 | 0.916295729 | 6.55E-05 | up |
| NDUFB10 | 1820482 | 1.467683024 | 6.64E-05 | up |
| PCBP2 | 6480411 | 1.296922763 | 6.64E-05 | up |
| RPS3A | 6380255 | 0.995648646 | 6.64E-05 | up |
| UBE4B | 2140563 | 0.851349469 | 6.64E-05 | up |
| WRB | 4590102 | 0.563758352 | 6.64E-05 | up |
| RAPGEF6 | 3390504 | -0.672882046 | 6.64E-05 | down |
| RPS15 | 5490603 | 1.512440234 | 6.76E-05 | up |
| MBNL2 | 990128 | 1.603622517 | 7.16E-05 | up |
| SDC2 | 2690026 | 1.600929972 | 7.16E-05 | up |
| CNPY2 | 1510564 | 1.281962896 | 7.16E-05 | up |
| MAGED2 | 3420487 | 1.247397333 | 7.16E-05 | up |
| RPS3A | 6560164 | 1.230142924 | 7.16E-05 | up |
| GOLT1B | 2100368 | 0.862688589 | 7.16E-05 | up |
| SUCLG2 | 4060692 | 1.144442877 | 7.65E-05 | up |
| C17orf79 | 3390477 | 0.920304871 | 7.65E-05 | up |
| RILPL1 | 5220309 | 1.001129512 | 8.42E-05 | up |
| RPS3A | 3180438 | 1.267085346 | 9.56E-05 | up |
| SNAPIN | 870041 | 0.448976723 | 0.000100663 | up |
| FASTK | 770687 | 0.914367321 | 0.0001063 | up |
| CAV2 | 5910553 | 0.879047002 | 0.0001063 | up |
| PRKRA | 770021 | 0.834038593 | 0.0001063 | up |
| FAM155A | 3710048 | 0.75233413 | 0.0001063 | up |
| YY1 | 610279 | 1.128257546 | 0.000107097 | up |
| COL8A2 | 70196 | 2.609508099 | 0.000107799 | up |
| DDAH1 | 3170292 | 1.740797333 | 0.00011324 | up |
| DAG1 | 2060091 | 0.840321903 | 0.000122272 | up |
| GABARAP | 6290132 | 0.673758241 | 0.000122272 | up |
| UBE3B | 5420025 | 0.321545224 | 0.000130534 | up |
| NELL1 | 1990731 | 2.608889501 | 0.000137633 | up |
| NMD3 | 4010048 | 1.069344286 | 0.000137633 | up |
| B9D1 | 6040465 | 0.44041778 | 0.000137633 | up |
| TMEM45A | 6280520 | 1.546856993 | 0.000138337 | up |
| RPL7 | 2680082 | 1.196436462 | 0.000152905 | up |
| C12orf62 | 6620360 | 0.75248323 | 0.000152905 | up |
| LRIG3 | 4540630 | 1.098188729 | 0.000158023 | up |
| THUMPD1 | 1740204 | 0.823367489 | 0.000158023 | up |
| VTI1B | 3890136 | 0.552409414 | 0.000158023 | up |
| ASNS | 1510296 | 2.035966022 | 0.00016527 | up |
| RPLP1 | 6060056 | 1.311855309 | 0.000166709 | up |
| RPS6 | 1410035 | 1.211362735 | 0.000170195 | up |
| EXOC4 | 2350215 | 0.435764545 | 0.000170195 | up |
| VPS37A | 1090221 | 0.558178834 | 0.00017261 | up |
| CYP2U1 | 4390541 | 0.437416746 | 0.000174377 | up |
| ANKRD33 | 4860707 | 1.600726163 | 0.000179869 | up |
| PSMC5 | 6580646 | 1.15069793 | 0.000183699 | up |
| B3GNT9 | 2900102 | 0.212076601 | 0.000183699 | up |
| RPS19 | 1470086 | 0.910551747 | 0.000184489 | up |
| RECK | 3390487 | 1.107484683 | 0.000184658 | up |
| TCEAL8 | 7550468 | 0.987193723 | 0.000186418 | up |
| GTPBP8 | 7000411 | 0.450939136 | 0.000186418 | up |
| EIF3A | 2060241 | 1.127068053 | 0.000190821 | up |
| SCCPDH | 5820187 | 0.185843873 | 0.000190821 | up |
| UBE2E1 | 2060465 | 0.994605658 | 0.000203176 | up |
| PRPF4 | 6100441 | -0.771910347 | 0.000203176 | down |
| SRPK2 | 2120731 | 0.337725148 | 0.000206822 | up |
| RABL2B | 7400376 | 0.152467518 | 0.0002139 | up |
| TMEM106C | 1430537 | 0.988603537 | 0.000217599 | up |
| UBE2Q2 | 830369 | 0.968712238 | 0.000217599 | up |
| TIMM8B | 540446 | 0.385336294 | 0.000217599 | up |
| SGCB | 1190162 | 0.647397494 | 0.000230243 | up |
| SLC27A2 | 4670138 | 2.715097599 | 0.000232674 | up |
| ADNP | 360678 | 0.620673111 | 0.000232674 | up |
| KRTCAP3 | 290435 | 0.185213204 | 0.000232674 | up |
| MARS | 2690707 | 0.917527185 | 0.000235295 | up |
| C20orf19 | 1300142 | 0.172488158 | 0.00023543 | up |
| RPN2 | 3370112 | 1.23717677 | 0.000242285 | up |
| XKR6 | 4730672 | 0.301251829 | 0.000251183 | up |
| TMEM33 | 6650382 | 0.286131257 | 0.000251183 | up |
| ASNS | 5960181 | 1.21213452 | 0.00025861 | up |
| GLT8D1 | 430400 | 0.93190902 | 0.00025861 | up |
| C14orf169 | 6510634 | 0.427600978 | 0.00025861 | up |
| CCT7 | 5270500 | 0.900292284 | 0.000261155 | up |
| EIF2S1 | 3870110 | 0.718457378 | 0.000261155 | up |
| ZNF511 | 1260435 | 0.634022341 | 0.000261155 | up |
| SERPINA5 | 3290630 | -2.897102352 | 0.000261155 | down |
| LOXL4 | 4150477 | -4.37749118 | 0.000261155 | down |
| NKIRAS2 | 6980164 | 0.777994696 | 0.000262293 | up |
| ADD1 | 1230289 | 0.352239499 | 0.000262293 | up |
| C16orf42 | 2370414 | 0.681662762 | 0.000262352 | up |
| TRMT112 | 5420398 | 1.354095863 | 0.000274973 | up |
| MATN2 | 670215 | 1.146050157 | 0.000274973 | up |
| MBNL2 | 2450546 | 0.713563059 | 0.000274973 | up |
| SNW1 | 830189 | 0.339186972 | 0.000274973 | up |
| PPA2 | 460630 | 0.970669946 | 0.000290895 | up |
| SF3B14 | 3780544 | 0.749713372 | 0.000295123 | up |
| CSNK1G2 | 1240192 | 1.889587571 | 0.000307324 | up |
| NACA | 6180066 | 1.025139993 | 0.000307324 | up |
| CLIP1 | 2260066 | 1.399269348 | 0.000316809 | up |
| GALNT10 | 3610202 | 1.363328282 | 0.000316809 | up |
| ALCAM | 2070575 | 1.325547308 | 0.000316809 | up |
| SAR1A | 3140553 | 1.158701298 | 0.000316809 | up |
| UBQLN2 | 5560482 | 0.919763373 | 0.000316809 | up |
| MATR3 | 3990189 | 0.899101444 | 0.000316809 | up |
| PNMA1 | 6350608 | 0.631028667 | 0.000316809 | up |
| BTD | 1410091 | 0.463450335 | 0.000319264 | up |
| UBE2D2 | 4760047 | 0.328764676 | 0.000324687 | up |
| ATF4 | 7610187 | 1.312436194 | 0.000332199 | up |
| ACVR2A | 4590349 | 0.636229597 | 0.00034269 | up |
| TRIB3 | 1990630 | 3.0873647 | 0.000343342 | up |
| TSPAN4 | 110661 | 1.455350419 | 0.000356479 | up |
| FAM179B | 4890082 | 0.379225944 | 0.000356479 | up |
| COPZ2 | 5820292 | 1.358023922 | 0.000356488 | up |
| TUSC3 | 3190474 | 0.920717232 | 0.000356488 | up |
| THBS3 | 1260039 | 2.781973195 | 0.000357009 | up |
| KCTD18 | 3140048 | 0.555572307 | 0.000357009 | up |
| GSTCD | 6130438 | 0.919218649 | 0.000357624 | up |
| SHC2 | 1410440 | 0.425332254 | 0.000363509 | up |
| TM2D1 | 10386 | 1.391795319 | 0.000366585 | up |
| KBTBD7 | 7210280 | 0.33983976 | 0.00038639 | up |
| P4HA1 | 4220731 | 1.793259975 | 0.000391664 | up |
| DUSP14 | 4150278 | 1.224222537 | 0.000391664 | up |
| RP6-213H19.1 | 3940286 | 1.965929369 | 0.000399966 | up |
| NPTN | 430307 | 1.244066088 | 0.000399966 | up |
| ATRN | 2320433 | 0.791117047 | 0.000399966 | up |
| TRPS1 | 6450484 | 0.760003076 | 0.000399966 | up |
| SH3YL1 | 2570288 | 0.442420339 | 0.000399966 | up |
| C10orf88 | 3440181 | 0.239869019 | 0.000401716 | up |
| CRNDE | 3780221 | 0.564967135 | 0.000418636 | up |
| RPL18AP3 | 510397 | 1.822821074 | 0.000418898 | up |
| CFDP1 | 6200494 | 0.683988899 | 0.000424018 | up |
| C17orf71 | 2490377 | 0.317138402 | 0.000424018 | up |
| ESYT3 | 610634 | -0.281352005 | 0.000432295 | down |
| DEPDC6 | 870390 | 1.152071526 | 0.000441453 | up |
| LMAN2 | 3850092 | 0.585966056 | 0.000443104 | up |
| MANBAL | 6840022 | 0.858837055 | 0.00044545 | up |
| LRPAP1 | 2650521 | 0.799748169 | 0.00044545 | up |
| RTCD1 | 7100193 | 0.58138579 | 0.000447391 | up |
| NOMO3 | 7210017 | 1.232661127 | 0.000451967 | up |
| UNC119 | 2940681 | 0.561559467 | 0.000470234 | up |
| TAPT1 | 610768 | 0.367649601 | 0.000470234 | up |
| PCTK3 | 1660025 | -0.532249167 | 0.000470234 | down |
| C2orf40 | 3140113 | -5.697453378 | 0.000470562 | down |
| TMEM47 | 2570730 | 1.866095365 | 0.000480656 | up |
| PCBP4 | 630274 | 0.791325532 | 0.000480656 | up |
| RAB15 | 5720392 | 0.234919392 | 0.000480656 | up |
| WDR20 | 3130494 | -0.456298637 | 0.00048238 | down |
| YTHDF3 | 4150240 | 0.754526546 | 0.000493303 | up |
| COPS8 | 3180020 | 0.872031108 | 0.000493882 | up |
| BACE1 | 2650414 | 0.374960529 | 0.000494216 | up |
| C17orf62 | 6560767 | -0.768762777 | 0.000499642 | down |
| LANCL1 | 4150138 | 0.875957604 | 0.000502492 | up |
| C12orf23 | 3290379 | 0.57788781 | 0.000502492 | up |
| R3HDM1 | 4860327 | 0.327654579 | 0.000502492 | up |
| UBE3C | 6560750 | 0.445069323 | 0.000503215 | up |
| TBC1D1 | 7510035 | 0.376718541 | 0.000510682 | up |
| TRIM56 | 2510239 | 0.600803325 | 0.000512532 | up |
| KIF1A | 6520440 | 1.82236059 | 0.000520018 | up |
| UBE2E2 | 5270021 | 1.022061269 | 0.000536233 | up |
| KTN1 | 4730148 | 1.356315729 | 0.000537972 | up |
| FNDC3A | 1660113 | 0.816247871 | 0.000537972 | up |
| CDC42SE2 | 6770161 | 0.882349524 | 0.000539937 | up |
| CAV1 | 5550379 | 1.45834034 | 0.000553325 | up |
| SMARCA2 | 5290474 | 0.758847956 | 0.000553325 | up |
| FRG1 | 2470519 | 0.556743307 | 0.000558277 | up |
| PRRC1 | 7040397 | 1.052568442 | 0.000559959 | up |
| COPS8 | 6940092 | 0.882660502 | 0.000559959 | up |
| SCUBE3 | 240070 | 0.937516328 | 0.000562152 | up |
| RPS16 | 6450553 | 0.423376108 | 0.000562152 | up |
| FGFR1OP2 | 1340681 | 0.408544655 | 0.00056374 | up |
| SS18L2 | 4760564 | -0.679021579 | 0.000565116 | down |
| ATXN1 | 520601 | 0.447982209 | 0.000567456 | up |
| RPL11 | 1070475 | 0.397169428 | 0.000573698 | up |
| CENPT | 6940554 | -1.15131505 | 0.000573698 | down |
| C14orf104 | 2490408 | 0.412178365 | 0.000575653 | up |
| C14orf166 | 3290689 | 1.129221365 | 0.00059325 | up |
| AGT | 2850301 | 0.709675808 | 0.00059325 | up |
| ING4 | 1430767 | 0.579139252 | 0.00059325 | up |
| CSNK1A1 | 3170468 | 0.55357109 | 0.00059325 | up |
| SLC33A1 | 4490470 | 0.400966892 | 0.00059325 | up |
| TRIM38 | 3930377 | -0.669344481 | 0.00059325 | down |
| WAC | 6660673 | 0.799975157 | 0.000613429 | up |
| FAM135A | 6560131 | 0.681900134 | 0.000613429 | up |
| IGFBP3 | 6590132 | 1.796059934 | 0.000614854 | up |
| PAPSS1 | 1820528 | 0.717936217 | 0.000614854 | up |
| BXDC5 | 2630626 | 0.532796368 | 0.000614854 | up |
| MED1 | 1070195 | 0.388611625 | 0.000614854 | up |
| PYCR1 | 2320161 | 0.275011132 | 0.000614854 | up |
| UNQ1887 | 2750612 | 0.312030498 | 0.000617456 | up |
| RPLP1 | 6380639 | 1.512794862 | 0.000626558 | up |
| MUSK | 4780653 | 0.27747196 | 0.00065251 | up |
| BAHD1 | 2650528 | 0.117807494 | 0.00065251 | up |
| LAPTM4A | 7050494 | 0.68132782 | 0.000661367 | up |
| YWHAG | 1770433 | 0.933441992 | 0.000676566 | up |
| TRIM2 | 7150240 | 0.811796288 | 0.000676566 | up |
| DEXI | 2470386 | 1.175128702 | 0.000693633 | up |
| TARS | 2710195 | 0.990775745 | 0.000693633 | up |
| MRPS35 | 7380270 | 0.582844598 | 0.000693633 | up |
| AVEN | 3800634 | 0.323776417 | 0.000693633 | up |
| ATG2B | 4780762 | 0.174866371 | 0.00069385 | up |
| VIM | 7050019 | 0.785676209 | 0.000704317 | up |
| APH1A | 7550364 | 0.406804781 | 0.000704317 | up |
| SNX14 | 4260504 | 0.630366796 | 0.000705451 | up |
| LRCH3 | 7210059 | -0.645464239 | 0.000706382 | down |
| MEGF8 | 1340598 | 0.400890028 | 0.000722827 | up |
| IFI27L2 | 1940274 | 1.084373557 | 0.000750774 | up |
| RPL3 | 1710369 | 0.729632916 | 0.000750774 | up |
| NAP1L1 | 6590484 | 0.36330416 | 0.000751044 | up |
| CTGF | 2640292 | 0.98838061 | 0.000756966 | up |
| UQCRC2 | 5220195 | 0.65141682 | 0.00075904 | up |
| GNB2L1 | 7650152 | 0.48374041 | 0.00075904 | up |
| RPL23 | 380575 | 1.533561874 | 0.000759707 | up |
| EDF1 | 3360471 | 1.064479376 | 0.000759707 | up |
| TXNDC9 | 4290521 | 0.431545895 | 0.000764427 | up |
| ASB8 | 870451 | 0.641477716 | 0.000766517 | up |
| CHMP2A | 4260762 | 0.679419523 | 0.000778104 | up |
| TMEM64 | 6270600 | 0.519238506 | 0.000778104 | up |
| SLC38A6 | 4010452 | 0.486900485 | 0.000778104 | up |
| TRA2B | 2490181 | 0.98095557 | 0.000778268 | up |
| HCFC1R1 | 50088 | 0.89417459 | 0.000778888 | up |
| YAP1 | 3360768 | 1.319747965 | 0.000779947 | up |
| LOC729317 | 990097 | 0.750102565 | 0.000788138 | up |
| C13orf37 | 1660181 | 0.471296891 | 0.000788138 | up |
| RRN3 | 4920403 | 0.33646273 | 0.000788138 | up |
| HARS2 | 3170519 | -0.836255647 | 0.000812571 | down |
| IFT52 | 520367 | 0.659924399 | 0.000814871 | up |
| CNIH4 | 6400332 | 0.933402715 | 0.00081725 | up |
| LOC653881 | 3990215 | 0.634062688 | 0.00081725 | up |
| SSPN | 3360373 | 1.37573743 | 0.000820194 | up |
| ATG5 | 2030228 | 0.633789455 | 0.000829416 | up |
| GTF2H1 | 7570343 | 0.281786067 | 0.000834643 | up |
| FBXL12 | 60768 | 0.262702027 | 0.000834643 | up |
| KLHL2 | 1070593 | 0.316554177 | 0.00084522 | up |
| DYNLRB1 | 4780047 | 0.999403821 | 0.000845284 | up |
| FNDC3A | 7200358 | 0.793735631 | 0.000858199 | up |
| WDR51B | 7160609 | 0.329669426 | 0.000858199 | up |
| PRTFDC1 | 940543 | 0.77746899 | 0.00086232 | up |
| C17orf39 | 3990736 | 0.304312237 | 0.00086232 | up |
| PSIP1 | 5310747 | 0.785820925 | 0.000876238 | up |
| 6-Sep | 6270020 | -0.385901008 | 0.00089614 | down |
| UBE4A | 520706 | 0.49575771 | 0.000901055 | up |
| HDHD2 | 6370477 | 0.652433711 | 0.00090108 | up |
| ACSL1 | 1030431 | 0.48351576 | 0.000914392 | up |
| ATF4 | 3460309 | 1.396221665 | 0.000924708 | up |
| CCT3 | 5270717 | 0.585537277 | 0.000924708 | up |
| IKIP | 3170605 | 0.585364669 | 0.000924708 | up |
| BAZ2B | 450075 | -1.050735163 | 0.000924708 | down |
| C19orf56 | 3800079 | 0.808546271 | 0.000944744 | up |
| GPX8 | 5720438 | 1.537033736 | 0.000946317 | up |
| STRAP | 4200672 | 1.194899099 | 0.000946317 | up |
| LOC729973 | 1230600 | 0.910179018 | 0.000951828 | up |
| WDR91 | 7150367 | -0.708057251 | 0.000959625 | down |
| PSMD13 | 7550601 | 0.416119922 | 0.000963217 | up |
| GMPR2 | 5090392 | 0.919155247 | 0.000967745 | up |
| SMARCA1 | 6200576 | 0.942432196 | 0.00098008 | up |
| UBA2 | 4290632 | 0.270840073 | 0.00098008 | up |
| RNF122 | 130593 | 0.344705518 | 0.000985139 | up |
| RAC1 | 6250300 | 0.245032336 | 0.000987378 | up |
| RGS22 | 6760561 | -0.479746905 | 0.000993648 | down |
| FAM134C | 2650010 | 0.279685328 | 0.000999359 | up |
| NUP54 | 4810358 | 0.600067658 | 0.001002537 | up |
| TF | 6590538 | -2.367202111 | 0.001007889 | down |
| IMPA1 | 6350360 | 0.91643279 | 0.001008555 | up |
| RPL7A | 6130079 | 1.018357072 | 0.001015323 | up |
| LAMP2 | 6480142 | 0.949367687 | 0.001015323 | up |
| EIF3M | 940398 | 0.497455827 | 0.001015323 | up |
| MRPS23 | 2680754 | 0.326678294 | 0.001015323 | up |
| DKC1 | 6980327 | -0.53442405 | 0.001015323 | down |
| MFSD8 | 7210762 | -0.660344015 | 0.001015323 | down |
| MRPL45 | 3710719 | 0.6758714 | 0.001019911 | up |
| CUL4A | 7200021 | 0.634870656 | 0.001019911 | up |
| MATR3 | 1710731 | -0.255188816 | 0.001019911 | down |
| CCDC85A | 5290026 | 1.380438957 | 0.00102116 | up |
| CCNG1 | 3170110 | 1.289787529 | 0.00102116 | up |
| XRCC5 | 2900039 | 0.785591397 | 0.00102116 | up |
| HNRPLL | 6560451 | 0.335794065 | 0.00102116 | up |
| PES1 | 1500066 | 0.307259856 | 0.00102116 | up |
| SRP72 | 2710386 | 0.631843363 | 0.001044033 | up |
| TRAPPC6B | 2680288 | 0.569218242 | 0.001076349 | up |
| C10orf72 | 1110201 | 0.230269565 | 0.001097337 | up |
| PDGFD | 5690139 | 1.998195039 | 0.001110352 | up |
| SNX4 | 7330014 | 0.953724805 | 0.001110352 | up |
| CARS | 20010 | 0.502066021 | 0.001110352 | up |
| TAF6 | 4050500 | 0.508017268 | 0.001117102 | up |
| PARN | 1440022 | 0.450196602 | 0.00112133 | up |
| CMPK1 | 2640551 | 1.123952348 | 0.001124605 | up |
| DR1 | 4890706 | 0.658779144 | 0.001149197 | up |
| MTMR6 | 160026 | 0.434574355 | 0.001153067 | up |
| UXS1 | 6760647 | 0.149997274 | 0.001153067 | up |
| AMPH | 360402 | 1.844467957 | 0.001167759 | up |
| TNFRSF10B | 2600463 | 1.365896419 | 0.001167759 | up |
| DCTD | 2480612 | 0.818437938 | 0.001169734 | up |
| NPNT | 5910398 | 0.753773633 | 0.001170462 | up |
| UGP2 | 150706 | 0.751207627 | 0.001175886 | up |
| RPL11 | 60053 | 0.286205399 | 0.001185372 | up |
| RFWD2 | 7570066 | 0.753561785 | 0.001191194 | up |
| TIRAP | 3180224 | 0.112384768 | 0.001191194 | up |
| FOXN3 | 4230470 | -1.048195869 | 0.001191194 | down |
| CDC16 | 4880521 | 0.502945717 | 0.001204695 | up |
| ZNF143 | 3830403 | -0.627143476 | 0.001204695 | down |
| UBR2 | 1990725 | -0.769268197 | 0.001204695 | down |
| ZNF146 | 3140167 | 0.737259627 | 0.00122375 | up |
| ANKRD27 | 5670112 | 0.190303638 | 0.001225911 | up |
| SHOC2 | 4590128 | 0.671348037 | 0.001255154 | up |
| PALM | 4540682 | 1.827314697 | 0.001267627 | up |
| HMGN2 | 4050541 | 1.811397565 | 0.001267627 | up |
| ZNHIT3 | 6650561 | 0.618302676 | 0.001267627 | up |
| TRAPPC6A | 6860072 | 0.48257694 | 0.001267627 | up |
| LCTL | 4780347 | 0.497717175 | 0.001267966 | up |
| TMEM93 | 3990600 | 0.998481679 | 0.001281634 | up |
| PRDX1 | 6250280 | 0.92166012 | 0.001281634 | up |
| LOC729009 | 3190133 | 1.112196915 | 0.001290395 | up |
| ZFR | 6060215 | 0.856199098 | 0.001290395 | up |
| ADD3 | 1990468 | 1.100688319 | 0.001298288 | up |
| AIM1 | 580255 | 0.381419672 | 0.001298595 | up |
| C16orf53 | 2340059 | 0.623897573 | 0.001330047 | up |
| SVIL | 3140603 | -1.288034294 | 0.001331544 | down |
| C19orf2 | 2600040 | 0.857209641 | 0.001335894 | up |
| FAM129A | 3370768 | 2.464251049 | 0.001349055 | up |
| KIF1B | 7040707 | -0.716783857 | 0.001349055 | down |
| THBS1 | 5810685 | 1.983142995 | 0.001349405 | up |
| ATP2B1 | 6510164 | 0.472345038 | 0.001349405 | up |
| C15orf44 | 4590468 | 0.271602678 | 0.001349405 | up |
| ARL5A | 70364 | 0.248543647 | 0.001349405 | up |
| NANOS1 | 2100367 | 0.158759019 | 0.001349405 | up |
| C10orf76 | 730593 | -0.440742356 | 0.001349405 | down |
| TEAD2 | 4670398 | -1.436244407 | 0.001349405 | down |
| RP6-213H19.1 | 2450152 | 1.842621359 | 0.001350438 | up |
| IQCC | 5270343 | 0.251266772 | 0.001350438 | up |
| BXDC2 | 1110722 | 0.577271924 | 0.001377446 | up |
| SNRPD1 | 1510039 | 0.299847071 | 0.001378146 | up |
| DDX49 | 450309 | 0.390920204 | 0.001380343 | up |
| CD59 | 1430240 | 0.150837045 | 0.001380343 | up |
| FBXO8 | 3420647 | 0.673340623 | 0.001387557 | up |
| DYRK4 | 6270382 | 0.669075426 | 0.001387557 | up |
| FUT8 | 3870497 | 0.642342652 | 0.001387557 | up |
| SMAD5 | 7560367 | 0.681044038 | 0.001395533 | up |
| SH3RF1 | 1410358 | 0.911977119 | 0.001410823 | up |
| VKORC1 | 6450546 | 1.257475499 | 0.001413234 | up |
| PTCHD1 | 6860176 | 0.869786009 | 0.001413234 | up |
| ASB1 | 1820347 | 0.827680983 | 0.001413234 | up |
| MYO5B | 1440014 | 0.16577081 | 0.001413234 | up |
| ZNF292 | 2630722 | -0.434554986 | 0.001413234 | down |
| KIAA0391 | 6560427 | -0.798979479 | 0.001414708 | down |
| LOC729009 | 6180070 | 0.935031356 | 0.001421966 | up |
| METTL10 | 6370075 | 0.205022384 | 0.001421966 | up |
| CNOT1 | 6520382 | -0.743470559 | 0.001421966 | down |
| MTX2 | 2480044 | 0.579553388 | 0.001430482 | up |
| CTDSPL | 1770170 | 0.963415302 | 0.001431817 | up |
| YWHAQ | 3850131 | 0.823146896 | 0.001431817 | up |
| DPYSL4 | 50309 | 1.777319452 | 0.001433258 | up |
| ZNF460 | 1260280 | 0.167914904 | 0.001445205 | up |
| ARMC5 | 2060154 | 0.377713099 | 0.001458832 | up |
| HSPA1A | 6380717 | 1.494206478 | 0.001459447 | up |
| NUMBL | 3520189 | 0.236169052 | 0.001487883 | up |
| PSMD10 | 5340370 | 0.279160421 | 0.001487907 | up |
| SLBP | 4280603 | 0.326669404 | 0.001500205 | up |
| DUSP11 | 4060523 | 0.097088815 | 0.001517578 | up |
| MEX3C | 6860445 | 0.415381586 | 0.001518171 | up |
| NUP54 | 6940201 | 0.276353255 | 0.001519922 | up |
| CIR1 | 2100136 | 0.669828875 | 0.00154167 | up |
| NME1 | 7560673 | 0.345199858 | 0.001545201 | up |
| SRP14P1 | 1300072 | 0.906114048 | 0.001584149 | up |
| RTN3 | 2470719 | 0.377267578 | 0.001584149 | up |
| SPATA7 | 3360609 | 0.146030353 | 0.00158973 | up |
| PRPS2 | 130619 | 0.617059746 | 0.001610119 | up |
| S100A13 | 5860148 | 1.406483145 | 0.001611679 | up |
| PLIN2 | 1400446 | -1.019723622 | 0.001620058 | down |
| C1QTNF4 | 4180091 | -1.731407999 | 0.001666043 | down |
| AURKAIP1 | 5810088 | 1.017420431 | 0.001679764 | up |
| PTPN12 | 3140364 | 0.943775358 | 0.001679764 | up |
| STUB1 | 4900431 | 0.925045282 | 0.001679764 | up |
| USP1 | 1170689 | 0.552784101 | 0.001679764 | up |
| WSB2 | 6550703 | 0.329263912 | 0.001679764 | up |
| SH3GLB2 | 7330259 | -0.997010077 | 0.001679764 | down |
| FAM129A | 4230735 | 2.333891162 | 0.00168002 | up |
| WFDC8 | 7330279 | 0.098059166 | 0.00168002 | up |
| SEC24A | 4880463 | 0.266688291 | 0.001689968 | up |
| CTU1 | 1230324 | 0.210347901 | 0.001689968 | up |
| MRPL48 | 730139 | 0.373674844 | 0.001704498 | up |
| USP33 | 650672 | 0.309170364 | 0.001706265 | up |
| ACVR1 | 6940360 | 1.251152985 | 0.001708856 | up |
| B4GALT2 | 4850082 | 0.253059463 | 0.001708856 | up |
| RRM2B | 1030639 | 0.157726476 | 0.001719531 | up |
| SMARCD3 | 5910632 | 1.229517042 | 0.001722538 | up |
| C16orf80 | 5360630 | 0.806168791 | 0.001724599 | up |
| MRPL24 | 3800445 | 0.494219558 | 0.001724599 | up |
| ATP2C1 | 3290279 | 0.142663872 | 0.001724599 | up |
| C1QTNF4 | 6520056 | -0.908249003 | 0.001724599 | down |
| ITGA6 | 2140678 | 0.497960108 | 0.001733033 | up |
| DNAJC10 | 4250452 | 1.243436767 | 0.001800737 | up |
| SLC11A2 | 1820037 | -1.079677517 | 0.001801251 | down |
| CDC42EP1 | 5420259 | 0.243830372 | 0.001805954 | up |
| LRBA | 6020291 | -0.586581757 | 0.001806886 | down |
| TCP1 | 4230136 | 0.24219258 | 0.0018133 | up |
| BTF3L4 | 1170717 | 0.922232828 | 0.001819066 | up |
| GDI2 | 6180538 | 1.077426774 | 0.001823489 | up |
| TMEM129 | 1450403 | 0.339409744 | 0.001823489 | up |
| ISCA1 | 5960221 | 0.614418784 | 0.001855707 | up |
| HSPA9 | 430630 | 1.113627969 | 0.001868351 | up |
| PGCP | 4210280 | 0.836054488 | 0.001891899 | up |
| TADA1L | 3840463 | 0.398669353 | 0.001943668 | up |
| ADO | 4480546 | 0.651370384 | 0.001948821 | up |
| CDV3 | 5720746 | 0.878762253 | 0.001971323 | up |
| SORCS3 | 870411 | 0.134528964 | 0.001971323 | up |
| HYDIN | 3390255 | -0.162178653 | 0.001971323 | down |
| C7orf47 | 1240592 | -0.530734143 | 0.001986563 | down |
| EIF4G2 | 2260095 | 1.481209834 | 0.002004191 | up |
| COL8A2 | 3780168 | 1.975369444 | 0.002010492 | up |
| IGFBP3 | 6840372 | 1.573315115 | 0.002010492 | up |
| AKAP11 | 1450333 | 0.799289472 | 0.002010492 | up |
| FTHL3P | 6450139 | 0.67339296 | 0.002010492 | up |
| ABCD1 | 4890609 | -0.239576773 | 0.002010492 | down |
| PLIN2 | 460204 | -1.410516604 | 0.002010492 | down |
| UBL5 | 6420541 | 0.949593974 | 0.002021089 | up |
| MAP3K12 | 4920300 | 0.219521031 | 0.002064109 | up |
| PAPOLA | 6940719 | 0.84269821 | 0.002073032 | up |
| PDE6D | 1500491 | 0.533846385 | 0.002088035 | up |
| USP3 | 6220477 | -0.968651852 | 0.002088035 | down |
| RAB6B | 4780463 | 0.662032208 | 0.002105705 | up |
| ABCA3 | 430402 | 0.396865752 | 0.002115062 | up |
| SMYD4 | 6350240 | -0.534759877 | 0.002130978 | down |
| TMEM200A | 4780523 | 0.909153504 | 0.002131218 | up |
| SENP3 | 870709 | 0.151011676 | 0.00214584 | up |
| ANKRD28 | 360064 | 0.32493189 | 0.002157435 | up |
| FAM120A | 430519 | 0.928649956 | 0.002157836 | up |
| SMNDC1 | 6520605 | 0.53799752 | 0.002157836 | up |
| SIRT1 | 7000224 | 0.47301224 | 0.002159079 | up |
| SEC31A | 5560180 | 0.918271532 | 0.002162613 | up |
| RNPEPL1 | 4230445 | 0.167109303 | 0.002162613 | up |
| RAB5A | 670367 | 0.878047589 | 0.002163093 | up |
| FARP1 | 5220689 | 0.097726245 | 0.002167533 | up |
| MSH5 | 3930414 | -0.348271076 | 0.002167533 | down |
| FOSB | 7160239 | 4.577903408 | 0.002170218 | up |
| ANXA2P3 | 2810411 | 0.185459622 | 0.002186199 | up |
| TSEN34 | 5820528 | 0.937591385 | 0.00218943 | up |
| C20orf30 | 1110541 | 0.860380777 | 0.00218943 | up |
| MAPKAPK5 | 3140156 | 0.455126415 | 0.00218943 | up |
| EIF1B | 6060414 | 0.936252581 | 0.002205431 | up |
| HEXIM1 | 4490286 | 0.163313267 | 0.002209191 | up |
| S100PBP | 6550086 | 0.355048636 | 0.002226531 | up |
| ME1 | 3390326 | 0.844191166 | 0.002230539 | up |
| RANBP1 | 4610047 | 0.797017811 | 0.002230539 | up |
| C5orf28 | 6200300 | 0.214196067 | 0.002230539 | up |
| SLC15A4 | 6650465 | -0.551663313 | 0.002230539 | down |
| FYTTD1 | 3180465 | 0.840950911 | 0.002231762 | up |
| POLR2H | 1240221 | 0.686576071 | 0.002233618 | up |
| PLA2G12A | 3310541 | 0.133497238 | 0.002235712 | up |
| PSENEN | 1110100 | 0.341599355 | 0.002237499 | up |
| SFPQ | 5050408 | 0.261748381 | 0.002253776 | up |
| ZNF384 | 1780176 | 0.221694419 | 0.002253776 | up |
| CIDECP | 1470253 | 0.211430305 | 0.002278567 | up |
| KLF10 | 3990292 | 0.540905765 | 0.002279517 | up |
| IRAK3 | 2360719 | -1.262845226 | 0.002296604 | down |
| ETS1 | 6370435 | 1.10873097 | 0.002307861 | up |
| MRPL19 | 2680541 | 0.212539703 | 0.002315418 | up |
| DDX56 | 7380653 | -0.763700773 | 0.002323632 | down |
| SP100 | 4200240 | -0.322938573 | 0.00234767 | down |
| TSPAN10 | 3310097 | 0.462240421 | 0.002354544 | up |
| FAM103A1 | 3930730 | 0.341289191 | 0.002378845 | up |
| YBX1 | 4150053 | 1.369778131 | 0.002406959 | up |
| PSMD9 | 7400725 | 0.334349123 | 0.002406959 | up |
| TM9SF3 | 2030270 | 0.738138153 | 0.002410888 | up |
| PNMAL1 | 6580400 | 1.197479875 | 0.002416153 | up |
| SGCB | 1510471 | 0.406307418 | 0.002452188 | up |
| IP6K1 | 6040064 | 0.775507289 | 0.002454046 | up |
| USP38 | 7000379 | 0.557580843 | 0.002456927 | up |
| RBM7 | 1340202 | 0.376195887 | 0.002456927 | up |
| EIF3CL | 5900255 | 0.166478966 | 0.002471025 | up |
| EXT2 | 6760070 | 0.818652808 | 0.002477153 | up |
| ADH5 | 1470035 | 0.426694897 | 0.0024806 | up |
| SKIV2L2 | 1450463 | 0.673677595 | 0.002495061 | up |
| MRPL15 | 4760520 | 0.69613457 | 0.002498459 | up |
| FAM10A4 | 4050195 | 0.64702805 | 0.002518355 | up |
| GNE | 6060209 | 0.689264763 | 0.002522115 | up |
| PECI | 10594 | 0.582576044 | 0.00254761 | up |
| ABHD15 | 5340450 | 0.286480512 | 0.00254761 | up |
| CD72 | 4200546 | -0.32707877 | 0.00254761 | down |
| 3-Mar | 4670255 | -0.870036011 | 0.002548542 | down |
| BCKDHB | 2140138 | 0.796478409 | 0.002565096 | up |
| CBX6 | 3370288 | 0.709391656 | 0.002565096 | up |
| RAP1B | 6590703 | 0.315141255 | 0.002583866 | up |
| TP53INP1 | 5420538 | 0.556703194 | 0.002589752 | up |
| TRIT1 | 7210092 | 0.553945377 | 0.002589752 | up |
| OR51F1 | 6100044 | -0.098833508 | 0.002589752 | down |
| APH1B | 7160224 | -0.678458527 | 0.002589752 | down |
| KIAA0649 | 3840193 | 0.238948892 | 0.002596883 | up |
| NANOS1 | 3180307 | 0.768383034 | 0.002598918 | up |
| UBLCP1 | 10278 | 0.614575088 | 0.002641238 | up |
| SET | 4230224 | 0.83454548 | 0.00264968 | up |
| COMT | 6940243 | 0.903974924 | 0.002662803 | up |
| MAGEH1 | 2490168 | 0.448389273 | 0.002683541 | up |
| C9orf82 | 380348 | 0.277656253 | 0.002688017 | up |
| 6-Sep | 7610370 | -0.650673676 | 0.002702236 | down |
| TMEM106B | 50427 | 0.990473803 | 0.002721634 | up |
| TCP1 | 990315 | 0.6379481 | 0.002759023 | up |
| ITIH3 | 6220025 | 1.688216732 | 0.002780822 | up |
| RPS11 | 1500538 | 1.206078329 | 0.002801208 | up |
| DEK | 4480180 | 1.200968786 | 0.002801208 | up |
| RPL37A | 990273 | 1.120662405 | 0.002808937 | up |
| PKN1 | 2360474 | 0.607847042 | 0.002808937 | up |
| ME3 | 1500743 | 0.136185836 | 0.002809378 | up |
| MCL1 | 610750 | 1.864284805 | 0.002810984 | up |
| CDC42 | 5270386 | 0.858208838 | 0.002810984 | up |
| PTPN13 | 3060609 | 0.586636248 | 0.002810984 | up |
| ATAD1 | 5340551 | 0.321523178 | 0.002858108 | up |
| TWISTNB | 5570343 | 0.314754055 | 0.002858108 | up |
| PUM1 | 3870543 | -0.382181068 | 0.002858108 | down |
| NGFRAP1 | 5260349 | 0.802956191 | 0.002872613 | up |
| SOCS5 | 6370379 | 0.512014995 | 0.002872613 | up |
| LDHB | 1400240 | 1.138181068 | 0.002872748 | up |
| SLTM | 4060136 | 0.970091621 | 0.002893487 | up |
| IFT74 | 3170722 | 0.418705191 | 0.002894087 | up |
| C9orf30 | 5260538 | 0.418081769 | 0.002904765 | up |
| GOLPH3 | 2320068 | 0.763288677 | 0.002905108 | up |
| HSPD1 | 70605 | 0.65930088 | 0.002905108 | up |
| ZNF629 | 5870687 | 0.395698128 | 0.002905108 | up |
| NR2C1 | 1470521 | -0.314030493 | 0.002906743 | down |
| CDKN2A | 5550671 | 0.274704571 | 0.002920367 | up |
| TTYH2 | 1690053 | -0.612320828 | 0.002920367 | down |
| PNMA2 | 4590619 | 1.457672602 | 0.002920652 | up |
| CALM2 | 7100711 | 1.268658138 | 0.002920652 | up |
| PRCP | 6110386 | 1.266590245 | 0.002920652 | up |
| COMT | 520446 | 0.590268918 | 0.002920652 | up |
| UBR7 | 6100576 | 0.511920851 | 0.002920652 | up |
| ATXN1 | 5080154 | 0.309771775 | 0.002920652 | up |
| TATDN1 | 1110167 | 0.281378179 | 0.002920652 | up |
| ZFYVE1 | 7210390 | 0.267630498 | 0.002920652 | up |
| BRD7 | 1570347 | 0.147907065 | 0.002920652 | up |
| PML | 2070682 | -0.098598626 | 0.002920652 | down |
| MED8 | 3060619 | 0.580657667 | 0.002941029 | up |
| ENAH | 1740161 | 0.333471513 | 0.002961904 | up |
| KCNMA1 | 5550452 | 1.661241313 | 0.002969588 | up |
| CARS2 | 160403 | 0.362836676 | 0.002969588 | up |
| CMTM4 | 2120634 | 1.236555602 | 0.002971302 | up |
| RAP2C | 4760438 | 0.50552593 | 0.002990444 | up |
| SEMA4B | 5080280 | -0.881268106 | 0.003043635 | down |
| MIF4GD | 1110243 | 0.605335693 | 0.003117549 | up |
| RNASEN | 4120022 | 0.40422129 | 0.003117549 | up |
| MRPS17 | 2690400 | 0.645311036 | 0.003118719 | up |
| NUP62 | 2510279 | 0.275447703 | 0.003154793 | up |
| SMARCD3 | 460463 | 0.755752748 | 0.003194791 | up |
| RANBP9 | 4610349 | 0.206673408 | 0.003196063 | up |
| ACSM3 | 6580735 | -0.517115346 | 0.003199768 | down |
| ATP2B1 | 4230564 | 0.332708869 | 0.003215267 | up |
| LOXL3 | 7200706 | 2.072960719 | 0.003235551 | up |
| LOC729009 | 1980594 | 1.015073359 | 0.003235551 | up |
| hCG_25371 | 1070731 | 0.938325597 | 0.003235551 | up |
| SOCS5 | 4880750 | 0.685552036 | 0.003235551 | up |
| CCPG1 | 60095 | 0.682044272 | 0.003235551 | up |
| YIPF5 | 380703 | 0.408354687 | 0.003235551 | up |
| ARNT | 2190040 | 0.354949307 | 0.003235551 | up |
| BLMH | 2850577 | 0.546213939 | 0.003236912 | up |
| KCTD1 | 430386 | 0.141964853 | 0.003236912 | up |
| TTC39C | 3310142 | -0.65560092 | 0.003236912 | down |
| SLC4A7 | 620543 | 0.735514631 | 0.00327052 | up |
| MGST1 | 5080131 | -1.111070482 | 0.00327052 | down |
| TAF7 | 430270 | 0.855858147 | 0.003287156 | up |
| SRRD | 5130487 | 0.330940789 | 0.003287156 | up |
| SARS | 6020093 | 1.208463357 | 0.003332525 | up |
| USP24 | 830634 | -1.075251517 | 0.003365917 | down |
| AMN1 | 6900044 | 0.162327646 | 0.003367708 | up |
| CDK7 | 4760369 | 0.615921871 | 0.003371916 | up |
| GLOD4 | 5910543 | 0.615374232 | 0.003371916 | up |
| DECR1 | 510291 | 0.909902672 | 0.003397801 | up |
| LRRC57 | 6760026 | 0.231795706 | 0.003406842 | up |
| HDAC4 | 5900468 | 0.284358645 | 0.003408357 | up |
| ATP5L | 4070544 | 0.547475004 | 0.003408424 | up |
| C9orf3 | 160307 | 0.973673193 | 0.00341294 | up |
| CP110 | 5260082 | 0.455333701 | 0.003415964 | up |
| MTDH | 3850154 | 0.868053218 | 0.003432659 | up |
| RABL5 | 5490484 | 0.363173672 | 0.003432659 | up |
| TRIP12 | 6590441 | 0.699516235 | 0.003437966 | up |
| PLEKHA1 | 6650324 | 1.390357089 | 0.003456667 | up |
| MYADM | 2970594 | 0.626026646 | 0.003459713 | up |
| STAMBPL1 | 7150059 | -0.928395457 | 0.003461349 | down |
| PALM | 3440037 | 0.859779578 | 0.003486134 | up |
| FAM45A | 990711 | 0.850677785 | 0.003486134 | up |
| RNF138 | 870563 | 0.599363252 | 0.003486134 | up |
| LZTFL1 | 6250154 | 0.576057502 | 0.003486134 | up |
| ZNF777 | 5860240 | 0.184471963 | 0.003486134 | up |
| STAG2 | 290458 | 0.166022926 | 0.003486134 | up |
| PBX3 | 2470634 | -1.269346374 | 0.003486134 | down |
| CHCHD7 | 3850180 | 0.157917995 | 0.003487372 | up |
| RPL23A | 4920408 | 0.611122097 | 0.00349463 | up |
| MOCS2 | 5550053 | 0.146721396 | 0.003517436 | up |
| GPM6A | 2690008 | 0.155401761 | 0.003519036 | up |
| SAP30L | 2320053 | 0.44644336 | 0.003521585 | up |
| NEK1 | 4760674 | 0.36833576 | 0.003587828 | up |
| MTX2 | 460072 | 0.862047684 | 0.003600112 | up |
| SBDS | 50170 | 0.791896621 | 0.003600112 | up |
| TUBA1A | 4490577 | 1.528193217 | 0.003662449 | up |
| GAS2 | 2900338 | 0.208476815 | 0.003662449 | up |
| C7orf28A | 3130477 | 0.332791601 | 0.003674108 | up |
| NUP205 | 1450524 | -0.514935792 | 0.003674108 | down |
| PFDN4 | 7200471 | 0.210172865 | 0.003674774 | up |
| NMD3 | 5360204 | 0.638095868 | 0.003674969 | up |
| SUMF1 | 5490626 | 0.580229963 | 0.003674969 | up |
| NCL | 3120458 | 0.520493387 | 0.003674969 | up |
| TBX1 | 460575 | 0.211771874 | 0.003674969 | up |
| C8orf59 | 1510452 | 0.950325922 | 0.003675048 | up |
| PRPSAP2 | 290112 | 0.422242773 | 0.003675048 | up |
| RPA4 | 3870717 | -0.391408628 | 0.003675048 | down |
| NIPA1 | 1190288 | 0.230912868 | 0.003696612 | up |
| SOCS6 | 4880475 | 0.121199968 | 0.003729438 | up |
| NARG2 | 1660687 | 0.320297559 | 0.003752603 | up |
| PHYH | 5860187 | 0.733781655 | 0.003779706 | up |
| INTS2 | 2000608 | 0.261591288 | 0.003780309 | up |
| LAMA4 | 5130435 | 0.503798326 | 0.003824896 | up |
| CHD6 | 4250082 | 0.242838238 | 0.00382732 | up |
| NDUFAB1 | 4860563 | 0.975130485 | 0.003850842 | up |
| SPHAR | 4010368 | 0.130279284 | 0.003850842 | up |
| SNF8 | 6520059 | 0.897581032 | 0.003851283 | up |
| DBNDD1 | 3870273 | 1.103373922 | 0.00390083 | up |
| RPS28 | 650349 | 1.26133757 | 0.003908321 | up |
| DMRTA1 | 6290717 | 0.74840113 | 0.003908321 | up |
| C14orf149 | 1300719 | 0.45569316 | 0.003908321 | up |
| MAGOH | 1450156 | 0.642577366 | 0.003930367 | up |
| ARSA | 4150168 | 0.4493833 | 0.003930367 | up |
| FAM8A1 | 4780768 | 0.417211247 | 0.003930367 | up |
| MED13L | 3190717 | 0.361219321 | 0.003942613 | up |
| ZFAND3 | 1400309 | 0.591880853 | 0.00394331 | up |
| ARMC1 | 7570209 | 0.510667116 | 0.00394331 | up |
| MRPL16 | 5310537 | 0.479380051 | 0.00394331 | up |
| SYTL3 | 2600682 | -0.413329225 | 0.00394331 | down |
| ORC2L | 6620475 | -0.615868092 | 0.00394331 | down |
| SMU1 | 3370661 | 0.367092134 | 0.003986206 | up |
| UBA1 | 150288 | -0.783932516 | 0.003986206 | down |
| CDIPT | 990161 | 0.362016463 | 0.00399948 | up |
| LBH | 2810246 | 1.23571804 | 0.004001726 | up |
| ARL8B | 130685 | 0.760003317 | 0.004003223 | up |
| PAK2 | 6220403 | -0.408919768 | 0.004067817 | down |
| RNF20 | 2630619 | 0.635689789 | 0.004131132 | up |
| PYCR1 | 6520681 | 1.194139108 | 0.004146295 | up |
| ALB | 650431 | -0.269903023 | 0.004183972 | down |
| NUAK1 | 7210497 | 0.947156616 | 0.00421001 | up |
| GRP | 160500 | 2.014335483 | 0.004234575 | up |
| QTRTD1 | 2490204 | 0.417353419 | 0.004234575 | up |
| EDN2 | 6400598 | -0.10254566 | 0.004234575 | down |
| EGFLAM | 5820402 | -1.343681672 | 0.004246297 | down |
| SP4 | 1010521 | 0.377046743 | 0.004303078 | up |
| LOC648603 | 1450093 | 0.519166003 | 0.004381736 | up |
| DBP | 7050458 | 1.067685381 | 0.004392687 | up |
| NUDT9 | 270341 | 0.272074886 | 0.004392687 | up |
| GTF3C4 | 1240014 | 0.103003555 | 0.004392687 | up |
| CLK1 | 3440138 | -1.64261589 | 0.004452002 | down |
| SPCS2 | 7040068 | 0.68550392 | 0.004469813 | up |
| KIAA0494 | 1090072 | 0.522770285 | 0.004469813 | up |
| CYP51A1 | 6200068 | 0.181520781 | 0.004474267 | up |
| LOC644037 | 4760243 | 0.983803016 | 0.004479011 | up |
| EHD2 | 5080523 | 0.771265494 | 0.004524042 | up |
| POLR2C | 6960546 | 0.518034285 | 0.004536714 | up |
| ATP5S | 2120100 | 0.215756207 | 0.004536714 | up |
| TGFBI | 7650358 | -1.680543655 | 0.004561033 | down |
| YIF1A | 4590494 | 1.108859141 | 0.00456611 | up |
| HSD3B7 | 1500608 | -0.544191007 | 0.00456611 | down |
| SYT7 | 6280356 | 0.089975073 | 0.004570152 | up |
| SCAP | 3140382 | 0.838292425 | 0.004583742 | up |
| GTF2IRD2 | 5560026 | -0.560686235 | 0.004583742 | down |
| CUL5 | 6060025 | 0.879820331 | 0.004622222 | up |
| RPS27A | 3780528 | 0.53805864 | 0.004632173 | up |
| ACAP2 | 60746 | 0.486303022 | 0.004632173 | up |
| RER1 | 3850431 | 0.381581017 | 0.004632173 | up |
| PRKCI | 3930561 | 0.147469123 | 0.004632173 | up |
| C21orf57 | 1110349 | 0.710229165 | 0.004678341 | up |
| RGMB | 5490181 | 0.131149599 | 0.004678341 | up |
| SLC40A1 | 5490601 | 0.571364524 | 0.004683347 | up |
| IFNA4 | 160762 | -0.088448933 | 0.004683347 | down |
| SQSTM1 | 4260044 | 0.912002983 | 0.00468493 | up |
| SFRS12 | 5900731 | 0.832775156 | 0.00468493 | up |
| OXSR1 | 4920669 | -0.817920474 | 0.00471178 | down |
| DCI | 2100079 | 0.774343171 | 0.004726789 | up |
| PLXNB2 | 10673 | 0.728859935 | 0.004733769 | up |
| VPS13D | 3290653 | -0.095952394 | 0.004785061 | down |
| PLCE1 | 6220687 | 0.745014725 | 0.004807155 | up |
| LRRC41 | 6590088 | 0.677716076 | 0.004807155 | up |
| DYNLRB1 | 7210224 | 0.565521273 | 0.004807155 | up |
| CD47 | 6270286 | 0.146372374 | 0.004807155 | up |
| DEDD | 1980220 | 0.133914569 | 0.004807155 | up |
| UQCRB | 520681 | 0.106365509 | 0.004807155 | up |
| HYDIN | 1780682 | -0.127979176 | 0.004807155 | down |
| CXorf57 | 6580626 | -0.875755029 | 0.004807155 | down |
| CAPN3 | 3870441 | -0.945318558 | 0.004807155 | down |
| PRKAR1A | 7050021 | 1.303787052 | 0.004811456 | up |
| ITFG2 | 6550195 | -0.602917749 | 0.004811456 | down |
| PDS5B | 870167 | 0.584177058 | 0.00482727 | up |
| KCTD16 | 5550315 | 0.244365465 | 0.00482727 | up |
| RTN4 | 780402 | 0.6378981 | 0.004830459 | up |
| ZC3H12B | 630195 | -0.541271248 | 0.004830459 | down |
| IER3IP1 | 6860681 | 0.58792468 | 0.004843318 | up |
| ZNF330 | 5260639 | 0.362264423 | 0.004843318 | up |
| TNC | 6940102 | 1.607865671 | 0.004847143 | up |
| FBXW7 | 5560661 | 0.58552463 | 0.004847143 | up |
| NAT15 | 4590451 | 0.503214395 | 0.004847143 | up |
| GPC1 | 7160504 | 1.731165663 | 0.0048954 | up |
| CMTM4 | 2230630 | 0.753719704 | 0.004919839 | up |
| UBR5 | 1340544 | 0.449193078 | 0.004919839 | up |
| ARID2 | 7100044 | 0.277294914 | 0.004924219 | up |
| ZNF451 | 2070189 | -0.592575796 | 0.004924219 | down |
| PGAP2 | 3710612 | 0.767452657 | 0.004930298 | up |
| ZNF16 | 730162 | 0.51988522 | 0.004930298 | up |
| DEPDC5 | 1090291 | -0.196768092 | 0.004930298 | down |
| NAP1L3 | 5270431 | 0.744951286 | 0.004940851 | up |
| SCOC | 4040482 | 0.890302823 | 0.00494135 | up |
| EWSR1 | 1230575 | 0.267135122 | 0.00494135 | up |
| UPF2 | 2750189 | -0.713423857 | 0.00494135 | down |
| GPKOW | 7200475 | 0.376796717 | 0.004942984 | up |
| NUP85 | 1430402 | -0.532131271 | 0.004942984 | down |
| C1orf26 | 3290017 | 0.142862024 | 0.004971689 | up |
| GRHPR | 7650615 | 0.581149453 | 0.004987113 | up |
| UBE2R2 | 5270291 | 0.14397222 | 0.005010701 | up |
| PLD3 | 6380093 | 0.727587729 | 0.00502409 | up |
| MED23 | 4900474 | 0.275672215 | 0.005029073 | up |
| SCRN2 | 7150647 | 0.276090424 | 0.005053408 | up |
| PKN3 | 4730170 | -0.668456888 | 0.00510032 | down |
| BAT1 | 160132 | -1.139003989 | 0.005151086 | down |
| PIGU | 3180554 | 0.420971349 | 0.005191014 | up |
| CLIC4 | 3890193 | 1.320156745 | 0.005193216 | up |
| FAM10A4 | 3610348 | 0.300607206 | 0.005229462 | up |
| SOX6 | 110735 | 0.141743542 | 0.005229462 | up |
| MGAT4B | 4060364 | 0.437609871 | 0.005263759 | up |
| AK3L1 | 4290192 | 0.63453357 | 0.005265128 | up |
| GNA13 | 6650161 | 0.273745859 | 0.005265128 | up |
| PLCB1 | 1570079 | 0.675985298 | 0.005268435 | up |
| ARPP19 | 2140368 | 0.433218871 | 0.005320884 | up |
| OTUD6B | 5550537 | 0.227731318 | 0.005320884 | up |
| TRAF3IP1 | 510528 | 0.140319136 | 0.005320884 | up |
| PGRMC2 | 1690326 | 1.017220422 | 0.005379529 | up |
| NR2F2 | 7380202 | 0.842369448 | 0.005379529 | up |
| RPL13 | 6060674 | 1.262225516 | 0.005392768 | up |
| ADIPOR2 | 6960685 | -0.560197111 | 0.00540078 | down |
| OSTC | 4610722 | 0.900424696 | 0.00542966 | up |
| KIAA0368 | 110678 | 0.65046122 | 0.00542966 | up |
| SEC24D | 4250451 | 0.596180445 | 0.00542966 | up |
| DNPEP | 5570754 | 0.294551166 | 0.005436144 | up |
| ARRDC2 | 3870504 | -0.320505528 | 0.005451463 | down |
| MRPL1 | 2680435 | 0.431235034 | 0.005452185 | up |
| MID1IP1 | 150438 | -0.63273613 | 0.005505733 | down |
| DMAP1 | 6040768 | 0.634342829 | 0.00555114 | up |
| RDH11 | 2340494 | 0.712385223 | 0.005570185 | up |
| CYB561 | 130086 | 0.525849847 | 0.005601095 | up |
| SS18 | 6380465 | 0.657842074 | 0.005615295 | up |
| CEP57 | 7100519 | 0.139233465 | 0.005629499 | up |
| CNOT8 | 4640484 | 0.688897542 | 0.005644974 | up |
| PDZRN3 | 3440681 | 0.142872469 | 0.005644974 | up |
| PSKH1 | 4060491 | 0.140332865 | 0.005778867 | up |
| SLC6A10P | 7610615 | -1.151609261 | 0.005783526 | down |
| HN1 | 4540435 | 0.292125396 | 0.005793042 | up |
| PWP2 | 2970133 | 0.099345637 | 0.005793042 | up |
| SUMO1P3 | 4050358 | 0.402571332 | 0.005820731 | up |
| UGDH | 4780050 | 0.859806583 | 0.005832999 | up |
| SCCPDH | 4490220 | 0.465431834 | 0.005832999 | up |
| SERPINB11 | 4290482 | -0.08427577 | 0.005837027 | down |
| AMY1A | 1820068 | 1.427518423 | 0.005852495 | up |
| KIF16B | 2900370 | 0.117154376 | 0.005852495 | up |
| SLC23A1 | 5090279 | 0.125510464 | 0.005854954 | up |
| MNAT1 | 2060564 | 0.392331565 | 0.005879923 | up |
| CDKAL1 | 2230020 | -0.326232883 | 0.005879923 | down |
| CCNY | 1340291 | 0.473342467 | 0.005890656 | up |
| EIF2AK2 | 2120079 | 0.815021737 | 0.005891492 | up |
| ZNF407 | 6400706 | -0.087332742 | 0.005937423 | down |
| PSAP | 7650333 | 0.589824883 | 0.005957207 | up |
| DEPDC6 | 3800095 | 1.204968589 | 0.005969106 | up |
| TMED10P | 1260129 | 0.959359501 | 0.005969106 | up |
| LOC644037 | 6510246 | 0.946604769 | 0.005969106 | up |
| ZBTB3 | 6200528 | 0.367406685 | 0.005969106 | up |
| NME1-NME2 | 1510609 | 0.322480265 | 0.005969106 | up |
| ZC3H7A | 1850288 | 0.169273207 | 0.005969106 | up |
| HEXDC | 3840370 | 0.135402947 | 0.005969106 | up |
| OPRL1 | 4860259 | 0.095917867 | 0.005969106 | up |
| UPF3B | 4640239 | -0.701956091 | 0.005969106 | down |
| PON3 | 4210228 | -1.923532582 | 0.005969106 | down |
| GBP2 | 1940162 | -2.441809127 | 0.005969106 | down |
| EIF4E2 | 4540632 | 0.808782436 | 0.00598623 | up |
| FNDC1 | 6900097 | 1.883981693 | 0.0059976 | up |
| CDK6 | 670286 | 1.881547135 | 0.00601239 | up |
| MRRF | 1980138 | -0.328079254 | 0.00601239 | down |
| COL5A3 | 5560300 | 0.340485273 | 0.006023079 | up |
| KLK3 | 460202 | 0.235797949 | 0.006039503 | up |
| TRIM47 | 840528 | 1.003530597 | 0.006064988 | up |
| 2-Sep | 6940619 | 0.204208883 | 0.006064988 | up |
| ATP8B2 | 6960168 | 1.092041466 | 0.006119656 | up |
| RAB6A | 4050349 | 0.131388463 | 0.006119656 | up |
| RPS29 | 7210372 | 0.350895541 | 0.006127455 | up |
| NIN | 2190196 | -0.438753609 | 0.006185105 | down |
| NOMO2 | 2340711 | 0.1073227 | 0.006213654 | up |
| DUSP6 | 5260288 | 0.865844126 | 0.006215057 | up |
| HDGFRP3 | 3460296 | 0.329140155 | 0.006215057 | up |
| CTBP1 | 7510538 | 0.682896746 | 0.006222528 | up |
| HLA-A | 3400438 | -0.674758101 | 0.006222528 | down |
| MSH5 | 6330440 | -0.244755393 | 0.006240845 | down |
| USP37 | 4150711 | -0.454986419 | 0.006353313 | down |
| SLC39A6 | 3060291 | 0.445329601 | 0.006356765 | up |
| XPO4 | 3800707 | 0.248498489 | 0.006356765 | up |
| POU5F1 | 5820538 | -0.36222111 | 0.006359205 | down |
| KCNG1 | 1400427 | 0.6365409 | 0.006395841 | up |
| PCGF6 | 1780437 | 0.16553318 | 0.006443845 | up |
| GRIK2 | 7210615 | 0.198804383 | 0.006447704 | up |
| SMTN | 6020441 | -1.324043478 | 0.006449408 | down |
| MRPL50 | 2060152 | 0.592273344 | 0.006499276 | up |
| SORCS2 | 4230739 | 0.824329483 | 0.006542959 | up |
| PISD | 1440278 | -0.372225045 | 0.00655369 | down |
| LOC727799 | 5290037 | 0.606077873 | 0.006588116 | up |
| ST3GAL5 | 5260403 | -1.326452811 | 0.006588116 | down |
| RPS14 | 5260682 | 0.500979618 | 0.006627927 | up |
| AFF3 | 650753 | 1.233133209 | 0.006690525 | up |
| PLCG1 | 1030376 | 0.2636333 | 0.006705276 | up |
| ATL3 | 1240243 | -0.699925975 | 0.006706119 | down |
| GHRL | 7320193 | -0.33248995 | 0.006742365 | down |
| C20orf134 | 7400170 | -0.258879382 | 0.006759827 | down |
| NUDCD2 | 610376 | 0.249191482 | 0.006790274 | up |
| C7orf49 | 7150259 | 0.369361914 | 0.006800153 | up |
| RBX1 | 2070746 | 1.067173274 | 0.006801141 | up |
| DISP1 | 5570246 | 0.692252715 | 0.006801141 | up |
| UBA6 | 2070019 | 0.44734067 | 0.006801141 | up |
| DACT1 | 3520241 | 0.685974135 | 0.006881534 | up |
| CREB3 | 7100520 | 0.301530934 | 0.006981979 | up |
| TRAPPC10 | 2970079 | 0.165405581 | 0.00700982 | up |
| DNAJC25-GNG10 | 540161 | 0.16444188 | 0.00700982 | up |
| STAG3L2 | 3710711 | -1.179663985 | 0.00715365 | down |
| TBL1XR1 | 540082 | 0.771006026 | 0.007166557 | up |
| PSMG1 | 2450136 | 0.497748216 | 0.007166557 | up |
| SLC3A2 | 4880161 | 0.458356114 | 0.007166557 | up |
| CEPT1 | 5870403 | 0.351085084 | 0.007166557 | up |
| LOC643668 | 270692 | 0.337654841 | 0.007166557 | up |
| TRMT61B | 2490170 | 0.285232625 | 0.007166557 | up |
| RPL39L | 6900672 | -0.59409894 | 0.007166557 | down |
| BTBD1 | 4230528 | 0.622436835 | 0.007192845 | up |
| KCNS3 | 5080259 | 0.13383619 | 0.007192845 | up |
| DLD | 1260195 | 0.623551598 | 0.007241079 | up |
| POU6F1 | 3140403 | 0.12903492 | 0.007241079 | up |
| USP42 | 6060040 | -0.199936536 | 0.007265451 | down |
| KIAA1199 | 110181 | 1.620206603 | 0.007274256 | up |
| NHP2 | 6550291 | 0.607433163 | 0.007274256 | up |
| TLK1 | 6770647 | 0.452940841 | 0.007274256 | up |
| BUB3 | 2100594 | 0.417600791 | 0.007274256 | up |
| RUSC2 | 2260577 | 0.914726651 | 0.007302191 | up |
| MYO5A | 1570056 | 0.703388838 | 0.007302191 | up |
| ZNF404 | 510719 | 0.521011762 | 0.007302191 | up |
| HDGF2 | 6110360 | 0.487158548 | 0.007302191 | up |
| BIVM | 1090367 | 0.428635669 | 0.007302191 | up |
| SERPINA11 | 50279 | 0.252870577 | 0.007302191 | up |
| ZSCAN1 | 2630593 | 0.154333873 | 0.007302191 | up |
| COX11 | 7560184 | 0.134153176 | 0.007302191 | up |
| C17orf47 | 6180082 | 0.119691499 | 0.007302191 | up |
| FRY | 4070553 | 0.097962826 | 0.007302191 | up |
| STAP2 | 5310132 | -0.173993334 | 0.007302191 | down |
| RHOBTB2 | 1090487 | 0.229407272 | 0.007315691 | up |
| DLEU1 | 1260612 | 0.218350922 | 0.007315691 | up |
| SHBG | 3800053 | 0.180455071 | 0.007315691 | up |
| SCAND2 | 2260296 | -0.478137778 | 0.007315691 | down |
| PRKAA1 | 6980750 | 0.482365332 | 0.007334493 | up |
| PSMD11 | 6220615 | 0.16736763 | 0.007338829 | up |
| COL11A1 | 5560739 | 2.852554694 | 0.007340222 | up |
| GALM | 2480450 | -1.071854181 | 0.007340222 | down |
| FH | 1780348 | 0.775861983 | 0.007369 | up |
| RPS13 | 3120717 | 0.556773496 | 0.0073753 | up |
| PANX2 | 4070300 | 0.819724238 | 0.007383851 | up |
| B4GALT4 | 6220672 | 0.267873527 | 0.007384943 | up |
| TM9SF2 | 5870632 | 1.168315504 | 0.007405086 | up |
| CHPT1 | 2260600 | 0.819728551 | 0.007405086 | up |
| DEGS1 | 5220632 | 0.761254574 | 0.007405086 | up |
| DAAM1 | 4490010 | 0.643238695 | 0.007405086 | up |
| RAB13 | 4390487 | 0.512214756 | 0.007405086 | up |
| RGS20 | 6400403 | 0.33507265 | 0.007405086 | up |
| SNRNP35 | 3840719 | 0.228151061 | 0.007405086 | up |
| TEX10 | 6770044 | -0.341334329 | 0.007405086 | down |
| PPAPDC3 | 5260278 | -0.765735729 | 0.007405086 | down |
| TSC1 | 4040373 | 0.675790163 | 0.007425383 | up |
| ZDHHC1 | 3370093 | 0.664166168 | 0.007465893 | up |
| FST | 1260068 | 0.983293755 | 0.007486787 | up |
| PPP2R3C | 5270138 | 0.506022738 | 0.007486787 | up |
| ZBTB44 | 5700020 | 0.135964325 | 0.007486787 | up |
| RNF4 | 2710541 | 0.330012836 | 0.007537978 | up |
| AZI2 | 5490348 | 0.562161016 | 0.007539919 | up |
| PAPSS1 | 130187 | 1.188134091 | 0.007557532 | up |
| CD63 | 3520167 | 1.141825837 | 0.007558849 | up |
| SLC24A1 | 4180689 | 0.180724527 | 0.007558849 | up |
| STAMBP | 1740364 | 0.592531003 | 0.007567347 | up |
| DDX50 | 430577 | 0.42959777 | 0.007584837 | up |
| PKIG | 6270088 | 0.211426034 | 0.007584837 | up |
| MANEAL | 3850685 | 0.18488787 | 0.007584837 | up |
| 3-Mar | 6760167 | -0.923153992 | 0.007584837 | down |
| METRN | 3120544 | 0.984487697 | 0.007640893 | up |
| CHCHD2 | 4540730 | 0.776839021 | 0.007647922 | up |
| EPB41L3 | 1510538 | 1.162247521 | 0.007680547 | up |
| SPIN2B | 6960543 | 0.108090324 | 0.007680547 | up |
| NR3C1 | 5820204 | 0.668821411 | 0.007730546 | up |
| CAMKK2 | 6550382 | 0.234819499 | 0.007730546 | up |
| PTPRE | 2490240 | -1.19206013 | 0.007730546 | down |
| CCDC34 | 3450408 | 0.601155427 | 0.007757642 | up |
| UBQLNL | 840360 | -0.435472891 | 0.007805891 | down |
| PIK3C3 | 6560121 | 0.172504557 | 0.007834112 | up |
| C10orf57 | 1170398 | 0.606226207 | 0.007841093 | up |
| KHDC1 | 6400088 | 0.205051568 | 0.007841566 | up |
| TMEM132D | 7400647 | -0.07089289 | 0.007841566 | down |
| INTS4 | 2810121 | -0.393097569 | 0.007841566 | down |
| SLC12A8 | 3710075 | 0.279030986 | 0.00784374 | up |
| UBE2H | 6020736 | 0.788468233 | 0.007860524 | up |
| AKTIP | 5720703 | 0.356707833 | 0.007897286 | up |
| CSDE1 | 3710440 | 0.419400005 | 0.007902148 | up |
| GRP | 1400053 | 1.134742398 | 0.007933507 | up |
| SOCS3 | 4230102 | 2.06298117 | 0.007982348 | up |
| TWSG1 | 940041 | 1.363301038 | 0.008025153 | up |
| GANAB | 2650446 | 0.648163551 | 0.008025153 | up |
| ZC3H15 | 4060484 | 0.565994276 | 0.008040194 | up |
| NCOR1 | 270646 | -0.495255433 | 0.00804914 | down |
| PSMA3 | 5720327 | 0.640933643 | 0.008055121 | up |
| PM20D2 | 50470 | 0.234456927 | 0.008070939 | up |
| TOMM5 | 5310241 | 0.828641448 | 0.008150725 | up |
| XRCC6 | 430100 | 0.724359069 | 0.008150725 | up |
| LOC100131294 | 1110450 | 0.554054068 | 0.008150725 | up |
| AZIN1 | 4070661 | 0.369400356 | 0.008150725 | up |
| PRR3 | 3930754 | 0.291747783 | 0.008150725 | up |
| PARP9 | 3140707 | 0.193626073 | 0.008150725 | up |
| SART3 | 4830717 | 0.18222203 | 0.008150725 | up |
| NCOA3 | 1450669 | 0.087746032 | 0.008150725 | up |
| STAG3L2 | 2070735 | -0.980814856 | 0.008150725 | down |
| AGAP6 | 770424 | -1.142425096 | 0.008150725 | down |
| RASL11A | 5910291 | 0.514769181 | 0.00815327 | up |
| ZNF193 | 4830220 | -0.502799302 | 0.008175873 | down |
| KIAA1267 | 1580338 | 0.520838292 | 0.008192267 | up |
| TMEM188 | 5340148 | 0.626759249 | 0.00820855 | up |
| FADS3 | 1230132 | 0.204741316 | 0.008252922 | up |
| RCN1 | 1500168 | 1.083647625 | 0.008287478 | up |
| PARD6A | 460086 | 0.235142831 | 0.008315631 | up |
| PRPS2 | 7000521 | 0.578339519 | 0.008324314 | up |
| WBP1 | 6380537 | 0.444339014 | 0.008324314 | up |
| SRC | 5420592 | -0.823344173 | 0.008324314 | down |
| FOXF2 | 1660470 | 0.458700589 | 0.008331821 | up |
| TRAF6 | 6330026 | -0.245792684 | 0.008331821 | down |
| OSBPL2 | 4180717 | 0.461302904 | 0.008346406 | up |
| NDRG4 | 7330739 | -0.902106214 | 0.008346406 | down |
| SUSD4 | 6020070 | 0.901540617 | 0.008371287 | up |
| DHX36 | 240646 | 0.368952692 | 0.008435686 | up |
| EFNB3 | 2680070 | 0.933053562 | 0.008446512 | up |
| ITGBL1 | 5570129 | 0.22528788 | 0.008446512 | up |
| ABCD3 | 1580736 | 0.340248941 | 0.008470286 | up |
| SRCAP | 7510687 | 0.114784426 | 0.008519093 | up |
| TTC39B | 2340477 | -0.226688953 | 0.008528097 | down |
| PRAMEL | 2810142 | -0.13392945 | 0.008550419 | down |
| MAP3K4 | 7320731 | -0.910698358 | 0.008615329 | down |
| IRAK1 | 4040564 | 1.002586139 | 0.008678231 | up |
| SEC24B | 150048 | 0.356970885 | 0.008678231 | up |
| SNURF | 4670601 | 0.197797937 | 0.008678231 | up |
| FIGNL1 | 6200634 | 1.565128215 | 0.008724518 | up |
| WARS | 3710068 | 1.202073212 | 0.008724518 | up |
| SLC2A6 | 6180465 | -0.327234981 | 0.008724518 | down |
| PTGES | 1510181 | -1.370907606 | 0.008724518 | down |
| ALG8 | 4850632 | -0.46588204 | 0.008735703 | down |
| TMEM41B | 1230546 | 0.969344385 | 0.008741333 | up |
| ATP5S | 6900722 | 0.259128428 | 0.0087641 | up |
| STAT1 | 2570079 | -0.729961384 | 0.008767482 | down |
| IFNA8 | 630041 | 1.166021226 | 0.008801741 | up |
| ZNF428 | 1820543 | 1.045083595 | 0.008801741 | up |
| RPS20 | 3360228 | 0.703389764 | 0.008801741 | up |
| SH2B2 | 3610390 | -0.212119535 | 0.008801741 | down |
| APPL2 | 4180100 | -0.652013043 | 0.008801741 | down |
| MCM6 | 5690274 | -0.759790955 | 0.008801741 | down |
| TSEN15 | 3870112 | 1.052602294 | 0.008874562 | up |
| PINK1 | 2490259 | 0.717791665 | 0.008874562 | up |
| P4HA2 | 4010064 | 0.712420053 | 0.008896268 | up |
| TP53I13 | 1010647 | 0.829680853 | 0.008898322 | up |
| YES1 | 2350397 | 0.536670431 | 0.008898322 | up |
| ZNF281 | 1240064 | 0.319549113 | 0.008898322 | up |
| TRAM2 | 940670 | 1.110332697 | 0.008913214 | up |
| GOPC | 1410170 | 0.360996297 | 0.008917197 | up |
| CAND1 | 240162 | 0.453561587 | 0.008926084 | up |
| CALY | 940746 | 0.402135177 | 0.008926084 | up |
| PTPRE | 2340673 | -0.925132414 | 0.008931237 | down |
| KCNRG | 6860634 | -0.219623649 | 0.008946171 | down |
| MST1 | 3710202 | -0.91355357 | 0.008946171 | down |
| ORMDL1 | 3170452 | 0.946407916 | 0.008962884 | up |
| NIPBL | 2360112 | 0.208967232 | 0.008962884 | up |
| TMEM67 | 1510373 | 0.096069884 | 0.008962884 | up |
| KLF16 | 3170301 | 0.083357668 | 0.008962884 | up |
| ZBTB8OS | 1500364 | 0.200006448 | 0.008970807 | up |
| CHCHD2 | 2120066 | 1.15392413 | 0.00898276 | up |
| PIWIL4 | 4540333 | -0.781552594 | 0.009038845 | down |
| ARPP-21 | 4560301 | 0.069931689 | 0.009069359 | up |
| EYA4 | 2350139 | 0.459242078 | 0.009089848 | up |
| ACOT11 | 1050253 | -0.647296987 | 0.009153168 | down |
| BDH1 | 5490593 | -0.391308183 | 0.009164259 | down |
| FAM69B | 4290152 | 0.115202399 | 0.009168805 | up |
| UCKL1 | 3930086 | -0.568700466 | 0.009183659 | down |
| PNPLA4 | 6560634 | 0.172722966 | 0.0092009 | up |
| NR2C1 | 270544 | 0.175568137 | 0.009206631 | up |
| WDR33 | 1030201 | -0.781079103 | 0.009209413 | down |
| RBPMS | 1240291 | 0.442035456 | 0.009230212 | up |
| GLT8D1 | 1710551 | 0.189252848 | 0.009230212 | up |
| ABLIM1 | 2570112 | -1.336016776 | 0.009230212 | down |
| FUSIP1 | 290687 | -0.594148443 | 0.009234129 | down |
| NIN | 1430376 | -0.330911903 | 0.009239229 | down |
| LMO4 | 7210450 | 1.770639302 | 0.009243644 | up |
| RIF1 | 1660646 | 0.313499696 | 0.009243644 | up |
| RTTN | 6400632 | -0.601058625 | 0.009243644 | down |
| RASSF1 | 2940739 | -1.154357174 | 0.009243644 | down |
| ROD1 | 6370678 | 0.841122379 | 0.009272598 | up |
| HSPE1 | 110110 | 0.509965045 | 0.009310436 | up |
| BTBD10 | 4250544 | 0.625411048 | 0.009346426 | up |
| OR51T1 | 5900224 | 0.127616709 | 0.009351276 | up |
| SPEG | 1440500 | 0.094597403 | 0.009376195 | up |
| FKBP10 | 60681 | 0.849034257 | 0.009380335 | up |
| MDN1 | 3140731 | -0.237689709 | 0.009380335 | down |
| SALL3 | 5090288 | 0.309251698 | 0.009417648 | up |
| PRPF38A | 4390370 | 0.19901639 | 0.009417648 | up |
| ARHGAP28 | 510575 | 0.143184545 | 0.009417648 | up |
| C17orf37 | 10717 | 0.160613308 | 0.009419465 | up |
| RABGGTB | 3990482 | 1.021443407 | 0.009422251 | up |
| CDH11 | 2650709 | 2.032122661 | 0.009505677 | up |
| PPM1D | 5690554 | 0.431902774 | 0.009565306 | up |
| SEC61B | 3390390 | 1.013835459 | 0.009589578 | up |
| PPP1R14C | 5490437 | 1.011034719 | 0.009589578 | up |
| PPP1CC | 2760292 | 0.868534051 | 0.009589578 | up |
| CBLL1 | 4070608 | 0.534757413 | 0.009589578 | up |
| ACVR2A | 4260681 | 0.274702014 | 0.009589578 | up |
| EPM2A | 2750392 | 0.1921759 | 0.009589578 | up |
| APC | 5720722 | 0.081297662 | 0.009589578 | up |
| RNPC3 | 2970315 | 0.112277351 | 0.009622812 | up |
| PSAP | 6200086 | 0.545983578 | 0.009724493 | up |
| RPS24 | 830066 | 0.605481038 | 0.009743635 | up |
| CSTF3 | 650598 | -0.965748992 | 0.009743635 | down |
| CNOT6L | 4730619 | -0.157273805 | 0.00974369 | down |
| FTSJ3 | 4210600 | 0.248424946 | 0.009763516 | up |
| ZNF354A | 6060296 | 0.131341234 | 0.009763516 | up |
| CCDC146 | 4860133 | -0.349367683 | 0.009777146 | down |
| 4-Sep | 5900543 | 0.872465884 | 0.009823679 | up |
| MAP6 | 7610220 | 0.462990612 | 0.009905406 | up |
| NAE1 | 6510619 | 0.363567193 | 0.009905406 | up |
| IGDCC4 | 6480014 | 1.157327419 | 0.009918475 | up |
| HOXD13 | 3990397 | 0.723290633 | 0.009918475 | up |
| DGCR6L | 7200274 | 1.152196841 | 0.009923828 | up |
| HNRNPH3 | 4390022 | 0.581930497 | 0.009923828 | up |
| FAM160B2 | 2710601 | 0.432191861 | 0.009977227 | up |
| RPS3A | 2640255 | 0.376643806 | 0.009977227 | up |
| IL19 | 6330070 | -0.089343729 | 0.009977227 | down |
| PPPDE1 | 3870255 | 0.614412035 | 0.009979155 | up |
| RAB18 | 1300768 | 0.393131058 | 0.009998781 | up |
| AK3 | 670463 | -0.45990568 | 0.009998781 | down |
